# Aqueous Keto-Polyethylene Dispersions from Catalytic Copolymerization of Ethylene and Carbon Monoxide in Water

**DOI:** 10.1021/acsmacrolett.4c00313

**Published:** 2024-06-24

**Authors:** Maximilian Baur, Rosa Habé, Stefan Mecking

**Affiliations:** Chair of Chemical Materials Science, Department of Chemistry, University of Konstanz, 78464 Konstanz, Germany

## Abstract

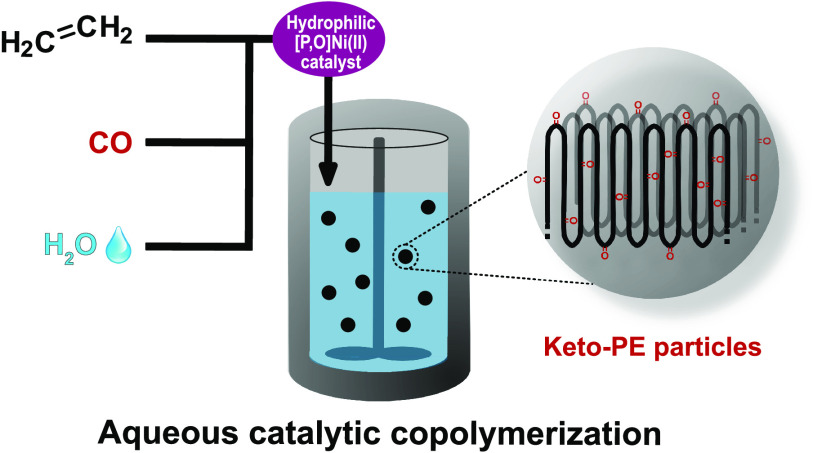

Water-soluble [P,O]Ni(II) catalysts enable the direct catalytic nonalternating copolymerization of fundamental comonomers ethylene and carbon monoxide (CO) in water as an environmentally friendly reaction medium. This yields stable aqueous dispersions of high molecular weight polyethylene containing ∼1 mol % of largely isolated in-chain keto groups in the form of particles with sizes between 100 nm and 1 μm. The intermediate species of chain growth resulting from incorporation of polar comonomers are amenable to specific chain termination pathways in conjunction with water.

Polymer dispersions are common in industrial and consumer applications, where they are used in adhesives, coatings, or paints.^1^ Here, the use of water as a dispersion medium is desirable due to its low cost, availability, and environmental safety, as well as health aspects in production and application.^2^ Polyethylene (PE) is also applied in the form of aqueous particle dispersions, which are either synthesized by postpolymerization emulsification^3−6^ or directly by free radical ethylene polymerization in water as reaction medium.^7−10^

Catalytic polymerization of ethylene to HDPE dispersions is also well studied employing late transition metal based catalysts, which ultimately enabled living ethylene polymerization to disentangled UHMWPE dispersions.^11,12^ However, the efficient incorporation of functional groups into HDPE materials^13−17^ via aqueous catalytic copolymerization of fundamental polar comonomers has not been achieved to date and was limited to few reports employing acrylates in emulsion polymerization to low molecular weight copolymers.^18,19^ Here the incorporation of in-chain keto groups to otherwise inert polyethylene is particularly desirable, as these in-chain keto groups can provide photodegradability^20,21^ as well as potential routes for chemical deconstruction.^22^ Aqueous free radical copolymerization of ethylene and CO can yield particle dispersions of photodegradable but branched keto-LDPEs.^23^ Catalytic copolymerization of ethylene and CO in water, however, is limited to reports of alternating copolymerization, thus, yielding high-melting (*T*_m_ > 200 °C) polyketone lattices.^24−29^

We now report how advanced, water-soluble *k*^2^[P,O]-phosphinophenolate Ni(II) catalysts can be used for the direct catalytic and nonalternating copolymerization of ethylene and CO in water as reaction medium, which yields aqueous particle dispersions of in-chain keto-modified polyethylene (keto-PE).

The established *k*^2^[N,O]Ni(II) salicylaldiminato catalysts as state of the art catalysts in aqueous polymerizations,^11,12^ have been found to be unsuitable for the demanding nonalternating copolymerization of ethylene with carbon monoxide.^30^ Neutral *k*^2^[P,O]Ni(II) phosphinophenolate catalysts bearing bulky substituents, on the other hand, have been successfully applied in the synthesis of in-chain keto-functionalized HDPE materials.^30^ These advanced phosphinophenolate Ni(II) catalysts derive from the traditional Ni catalysts employed in the *Shell Higher Olefin Process*, which were reported for catalytic ethylene polymerization in water early on, but produced only low molecular weight PE due to high rates of β-H elimination.^31,32^ This long-standing drawback could be revised by introducing bulky substituents to effectively suppress β-H elimination and enable high molecular weight PE.^33,34^

Recently, these advanced phosphinophenolate Ni(II) catalysts were also employed for living ethylene polymerization to UHMWPE in aqueous microemulsion,^35^ as well as in a permanently hydrophilic form.^36^ Such neutral, hydrophilic phosphinophenolate Ni(II) catalysts, obtained by sulfonation of the aryl substituent on the phosphine chelating ligand (Figure 1b), can catalyze nonalternating ethylene-CO copolymerization in water (vide infra). Additionally, novel phosphinophenolate Ni(II) catalysts bearing hydrophilic α-amino-polyethylene glycol (NH_2_-PEG) as a labile ligand can be used as an alternative approach to obtain a water-soluble catalyst precursor (Figure 1a). Such water-soluble catalyst precursors bearing hydrophilic labile ligands transform to a lipophilic active species upon activation^37^ and therefore rely on polymerization in surfactant micelles, as demonstrated in aqueous ethylene polymerization, employing neutral *k*^2^[N,O]Ni(II) salicyladiminato^11^ and *k*^2^[P,SO_3_]Pd(II)/Ni(II) phosphinosulfonate catalysts.^38,39^

**Figure 1:**
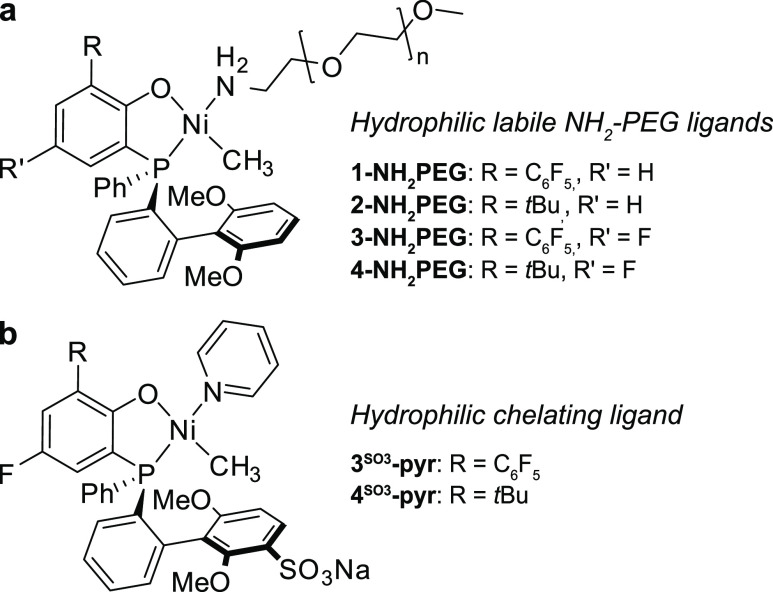
Water-soluble [P,O]Ni(II) phosphinophenolate catalyst precursors for aqueous nonalternating copolymerization of ethylene and CO. (a) Water-soluble complexes bearing a hydrophilic NH_2_-PEG labile ligand. (b) Permanently water-soluble complexes bearing a hydrophilic −SO_3_Na group at the bulky aryl substituent of the phosphine and pyridine as a labile ligand.

Previous insights on nonalternating ethylene/CO copolymerization^30,40^ anticipated high polymerization temperatures and low CO to ethylene concentrations in the fed monomer stream as favorable polymerization conditions to alleviate kinetic differences in monomer reactivity. Additionally, suitable reaction conditions were established with CsOH and sodium dodecyl sulfate (SDS), as previously reported for aqueous catalytic ethylene polymerization.^11,36^

Based on this rational, hydrophilic catalyst precursor **1-NH**_**2**_**PEG** was exposed to ethylene–CO gas mixtures containing between 0.6% and 2.0% CO at 90 °C (Table S1). While no CO incorporation was observable with a monomer feed composition of 0.6% CO, only traces of polymer was formed with 2% CO content in the monomer feed (*cf*. Table S1, entries 1 and 5). Monomer feeds containing between 1.0 and 1.4 mol %, on the other hand, successfully yielded polymer dispersions (Table S1, entries 2–4), even though polymerization activities and yields are substantially reduced by the presence of already small amounts of CO (Figure S10a).

Analysis of the obtained polymers revealed that these are indeed in-chain keto-functionalized polyethylenes with a distinctive C=O stretching absorption at 1716 cm^–1^, which is characteristic for predominantly isolated keto-groups in the PE matrix (Figure 2a).

**Figure 2:**
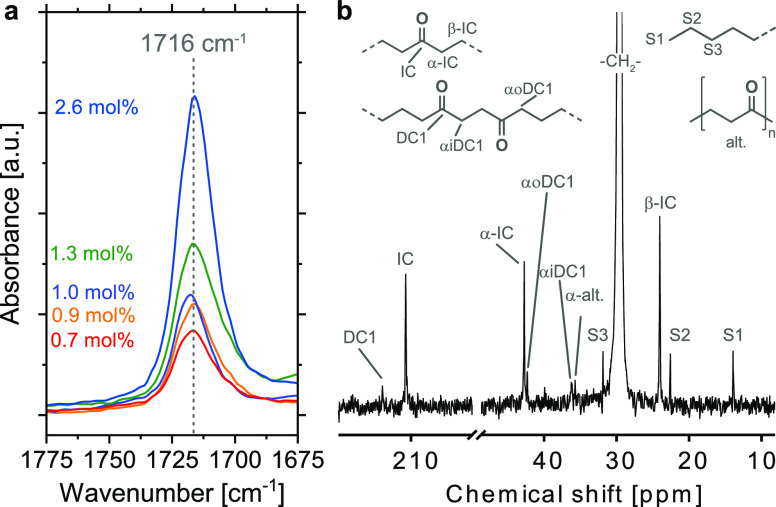
(a) Carbonyl region in ATR-IR spectroscopy (1675–1775 cm^–1^) of keto-PEs with different carbonyl contents obtained from aqueous nonalternating copolymerization of ethylene and CO. The characteristic C=O absorption band at 1716 cm^–1^ shows the predominantly isolated incorporation of CO into the PE chain. (b) ^13^C NMR spectroscopy (101 MHz, 383 K, C_2_D_2_Cl_4_) of a keto-PE obtained from aqueous catalytic copolymerization with complex **1-NH**_**2**_**PEG** (Table S1, entry 3).

This was confirmed by ^13^C NMR spectroscopic analysis, which also showed characteristic resonances for prevailing isolated in-chain keto-groups in a polyethylene chain with a carbonyl peak at 210 ppm (Figure 2b and Supporting Information). Molecular weights of the obtained copolymers were lowered compared to aqueous ethylene homopolymerization (*cf.*Table S3) and decrease with higher CO contents in the monomer feed (Table S1, entries 1–4). This observed trend of reduced molecular weights in CO copolymerization vs ethylene homopolymerization is opposite to observations made for nonaqueous polymerizations, where generally higher molecular weights were observed in the presence of CO due to blocking of free coordination sites, required for β-H elimination.^30^ This indicates a distinctive influence of the presence of CO on the chain transfer/termination reaction in the highly polar and protic reaction environment. For aqueous ethylene homopolymerization catalyzed by [N,O]Ni(II) salicylaldiminato or [P,O]Ni(II) phosphinophenolate complexes, the prevailing chain termination and catalyst decomposition pathway is protonolysis of the metal–alkyl species by H^+^ (Figure 3a) and high pH values of the reaction medium proved beneficial for catalyst stability and polymerization activity.^36,41^ In ethylene-CO copolymerization, on the other hand, intermediate metal-acyl species, formed after CO insertion, might rather be sensitive to a nucleophilic attack by OH^–^ due to the different partial polarizations (Figure 3b; Note that clear spectroscopic identification of formed carboxylic end groups would require isotopic labeling). Additionally, the chelates formed after successive CO and ethylene insertion^30,40^ can be sensitive for deprotonation (Figure 3c). This is indicated by the observation of α,β-unsaturated keto-end groups at ∼6.40 ppm in ^1^H NMR spectroscopic analysis of all copolymer samples (*cf.*Figures S21–S28), if compared to aqueous homopolymerization (Figure S32) and reported nonaqueous ethylene/CO copolymerization,^30^ in which no indications for such a pathway of chain termination were observable. Notwithstanding, also unfunctionalized olefinic end groups (H_2_C=CH-R) formed by chain transfer via β-H elimination from metal–alkyls were observed in ^1^H NMR spectroscopic analysis (*cf.*Figures S21–S27), which can occur in parallel to the aforementioned chain termination reactions.

**Figure 3:**
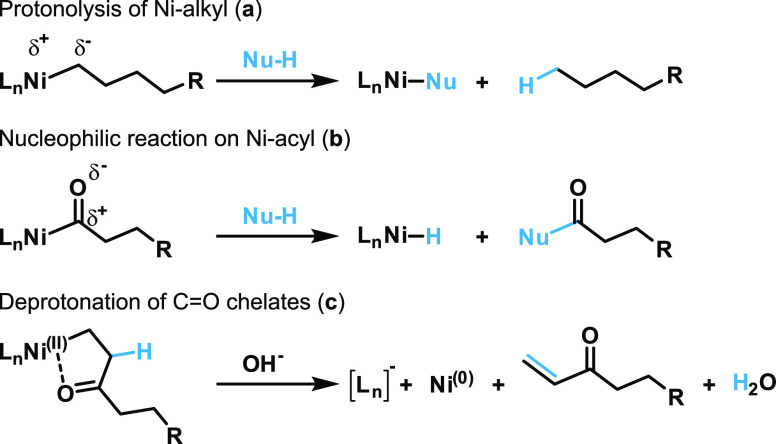
Potential pathways of different chain termination reactions in aqueous ethylene-CO copolymerization: Protonolysis of Ni-alkyl species (a), nucleophilic reaction on intermediate Ni-acyl species (b), and deprotonation of C=O chelates formed after successive CO and ethylene insertion (c).

Copolymerization experiments employing **1-NH**_**2**_**PEG** at varying pH values (Table 1) showed a distinctive influence of the OH^–^ concentration on copolymerization productivity (Figure 4a,b). While acidic pH values still resulted in reduced copolymerization yields, catalyst productivity showed a peak at a neutral pH value and decreased with higher OH^–^ concentrations (Figure 4a and Table 1). This is contrary to aqueous ethylene homopolymerizations, where higher catalytic productivity was observed at elevated pH values (Figure 4a and Table S3, entries 1–3) thus evidencing different pathways of chain termination in aqueous ethylene-CO copolymerization compared to ethylene homopolymerization.

**Table 1:** Results of Aqueous Ethylene/CO Copolymerizations with **1-NH**_**2**_**PEG**^a^

entry	pH	yield [g]	χ (CO)^b^ [mol %]	TOF^c^	*M*_n_ (*M*_w_/*M*_n_)^d^ [10^3^ g mol^–1^]	*T*_m_ [°C] (cryst. %)^e^	*Z*-avg [nm]^f^
1	2.2	no yield					
2	4.2	0.18	1.8	0.6	68 (1.5)	135 (70)	653 ± 49
3	7	0.47	0.9	1.7	80 (1.6)	134 (71)	1008 ± 92
4	9.8	0.28	1.3	1.0	57 (1.6)	133 (71)	748 ± 55
5	10.8	0.29	0.7	1.0	47 (1.6)	134 (73)	331 ± 7
6	11.8	0.23	0.9	0.8	40 (1.6)	134 (73)	241 ± 2
7	12.8	0.14	0.7	0.5	29 (1.5)	132 (72)	147 ± 3

a Polymerization conditions: 10 μmol, **1-NH**_**2**_**PEG**, 90 °C, 30 bar, 100 mL of oxygen free water, 750 mg sodium dodecyl sulfate (SDS), 1.2% CO/C_2_H_4_ in an automated gas feed, 1000 rpm pitched blade stirrer, 60 min.

b Carbon monoxide incorporation determined by IR spectroscopy (*cf.*Supporting Information for details).

c TOF given in units of 10^3^ mol [C_2_H_4_] mol^–1^ [Ni] h^–1^.

d Determined by SEC in 1,2-dichlorobenzene at 160 °C, 0.5 mL min^–1^ via universal calibration versus narrow polystyrene standards.

e Determined by DSC (10 K min^–1^), second heating cycle.

f Determined by dynamic light scattering.

**Figure 4:**
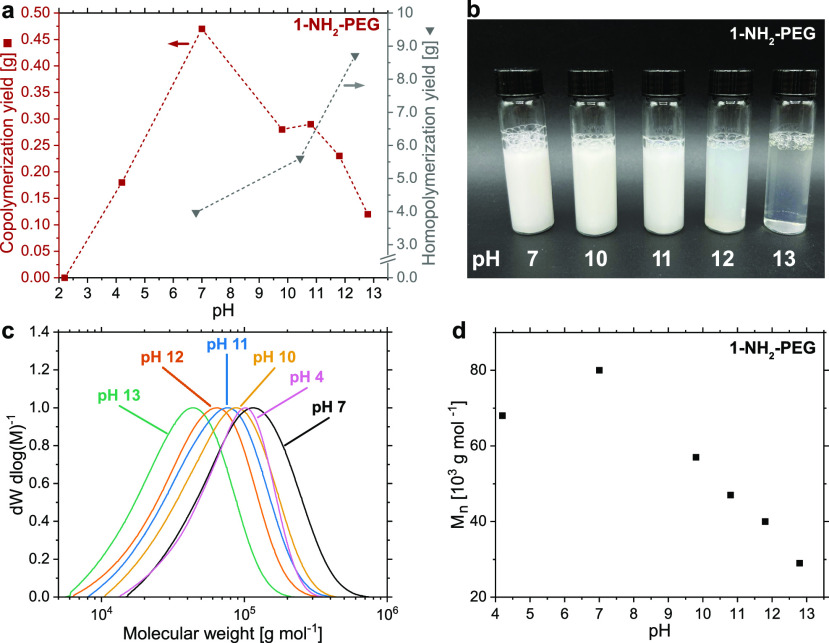
Influence of different pH values on aqueous catalytic ethylene-CO copolymerization with **1-NH**_**2**_**PEG** (*cf*. Table 1): (a) Copolymerization yields (red squares) in dependence of pH. Ethylene homopolymerization yields (gray triangles) for comparison. Dashed lines are only guides to the eye. (b) Aqueous keto-PE dispersions were obtained with **1-NH**_**2**_**PEG** at different pH values. (c) SEC traces (160 °C in 1,2-dichlorobenzene) of keto-PEs were obtained with **1-NH**_**2**_**PEG** at different pH values. (d) Development of the molecular weight (*M*_n_) of keto-PEs obtained at different pH values.

Such a proposed influence of the OH^–^ concentration on chain termination is supported by an increased ratio of the α,β-unsaturated keto-end groups in comparison to unfunctionalized olefinic (H_2_C=CH-R) end groups (Table S4).

Molecular weights of the obtained copolymers from aqueous copolymerization were also dependent on the pH value of the reaction medium (Figure 4b,c). The highest molecular weight was obtained at a neutral pH of ∼7 (*M*_n_ = 80 × 10^3^ g mol^–1^), while acidic (pH 4.2) and basic reaction conditions reduced the obtained molecular weights. In contrast to the observed dependence of copolymerization yields and molecular weights on the pH of the reaction medium, the CO incorporation in the copolymers remained around the targeted value of ∼1–2 mol % within experimental deviations even at different pH values (Figure S10b and Table 1, entries 2–7).

Following the successful aqueous nonalternating ethylene/CO copolymerizations with precatalyst **1-NH**_**2**_**PEG**, other water-soluble Ni(II)-phosphinophenolate complexes bearing NH_2_-PEG labile ligands **2-NH**_**2**_**PEG - 4-NH**_**2**_**PEG** (Figure 1a) as well as catalysts with a hydrophilic chelating ligand **3**^**SO3**^**-pyr** and **4**^**SO3**^**-pyr** (Figure 1b) were subjected to copolymerization conditions previously studied with **1-NH**_**2**_**PEG** (Table 1). Notably, all of the studied water-soluble phosphinophenolate Ni(II) catalysts yielded aqueous dispersions containing the desired keto-PE particles with varying incorporation ratios of CO between 0.7 and 2.6%, according to IR spectroscopy (*cf.*Figures 2 and S11–S19). Polymerization yields in copolymerizations with NH_2_-PEG substituted precatalysts **2-NH**_**2**_**PEG-4-NH**_**2**_**PEG**, as well as −SO_3_Na-substituted **4**^**SO3**^**-pyr**, however, were lower compared to yields obtained with **1-NH**_**2**_**PEG**. A general trend for slightly higher productivity of the complexes bearing a C_6_F_5_-substituent in *ortho* position to the oxygen on the phosphinophenolate ligand could be observed, which is in line with previous observations made in nonaqueous copolymerizations regarding catalyst productivities.^30^ Further, catalysts bearing a NH_2_-PEG hydrophilic labile ligand yielded generally higher molecular weights than hydrophilic catalysts bearing −SO_3_Na groups (Table S2, entries 2–20 vs 21–25, and Table S3).

Additionally, the molecular weights of the obtained copolymers with NH_2_-PEG complexes are generally lowered compared to their reference ethylene homopolymerization experiments (*cf.*Table S3), whereas the molecular weights of the copolymers obtained with hydrophilic −SO_3_Na catalysts are in a similar range compared to their PE homopolymers. In NMR spectroscopic analysis of copolymers obtained with *ortho*-C_6_F_5_-substituted, hydrophilic catalyst **3**^**SO3**^**-pyr** unfunctionalized olefinic end groups (H_2_C=CH-R) from common β-H elimination of polyethylene chain growth prevail (Figures S26 and S30). Therefore, β-H elimination is likely faster for catalyst **3**^**SO3**^**-pyr** compared to the NH_2_-PEG-substituted complexes and lower molecular weight copolymers are obtained for **3**^**SO3**^**-pyr** under otherwise comparable polymerization conditions (Table S2 and Figures S33–S35) with the molecular weights dominated by relatively high rates of β-H elimination from metal-alkyls rather than other chain-termination reactions. Consequently, the amount of polymer chains theoretically formed per catalyst added to the reaction mixture is higher for **3**^**SO3**^**-pyr**, *e.g.*, ∼9/[Ni-added] at pH 11.8 (Table S2, entry 24) vs ∼1/[Ni-added] for **1-NH**_**2**_**PEG** (Table 1, entry 6). Complex **3-NH**_**2**_**PEG**, bearing an *ortho*-C_6_F_5_ group, showed a very similar catalytic behavior and pH dependence (Table S2, entries 12–16) compared to **1-NH**_**2**_**PEG**, but suffers from an overall lower catalytic activity. Precatalysts **2-NH**_**2**_**PEG** and **4-NH**_**2**_**PEG** bearing *ortho-t*Bu substituents, on the other hand, showed a less straightforward trend of pH dependence with generally much lower catalyst stability and yields (Table S2, entries 8–11 and 17–20). Permanently hydrophilic catalysts **3**^**SO3**^**-pyr** (−C_6_F_5_) and **4**^**SO3**^**-pyr** (-*t*Bu) both showed a more complex pH dependence with a peak in catalytic activity at pH 11.8, most likely due to an influence of the ionic −SO_3_Na group (Table S2, entries 21–30). Even though clear trends beyond catalyst **1-NH**_**2**_**PEG** are less pronounced, the *ortho*-C_6_F_5_ substitution motif is generally superior to the *ortho-t*Bu substituents for obtaining the desired keto-PE dispersions in aqueous copolymerizations regarding the aspects of catalyst activity, stability, and robustness toward changes of polymerization conditions.

The keto-PE copolymers were obtained in the form of stable aqueous particle dispersions directly from polymerization (Figure 4, **b**) as the formed keto-PE particles are *in situ* stabilized by the added sodium dodecyl sulfate (SDS) surfactant. Analysis of the copolymer dispersions by dynamic light scattering (DLS, *cf.*Tables 1 and S2 and Figures 5a and S37) and transmission electron microscopy (TEM, Figures 5b, S38, and S39) showed predominantly spherical keto-PE particles in the range between ∼100–1000 nm (volume based *Z*-average), which are larger if compared to previous reports on Ni-catalyzed aqueous ethylene homopolymerization.^36^ The largest particles (*Z*-average ∼ 1000 nm) were obtained from copolymerizations at neutral pH ∼ 7 with precatalyst **1-NH**_**2**_**PEG** (Table 1, entry 3, Figure 5a). More acidic as well as more basic pH of the reaction medium led to a decrease in the observed particle sizes (*Z*-average) and also resulted in substantial broadening of the obtained particle size distributions (*cf.*Figure 5a and Table 1). These aqueous keto-PE particle dispersions could be successfully deposited by drop casting to form continuous keto-PE films with a thickness of ∼5 μm (Figures 5c and S40). Even though the high molecular weights and high crystallinities (>70%) of the keto-PEs resulted in rather brittle and slightly turbid films, this ability is nevertheless a key prerequisite for potential applications in coatings or paints.

**Figure 5:**
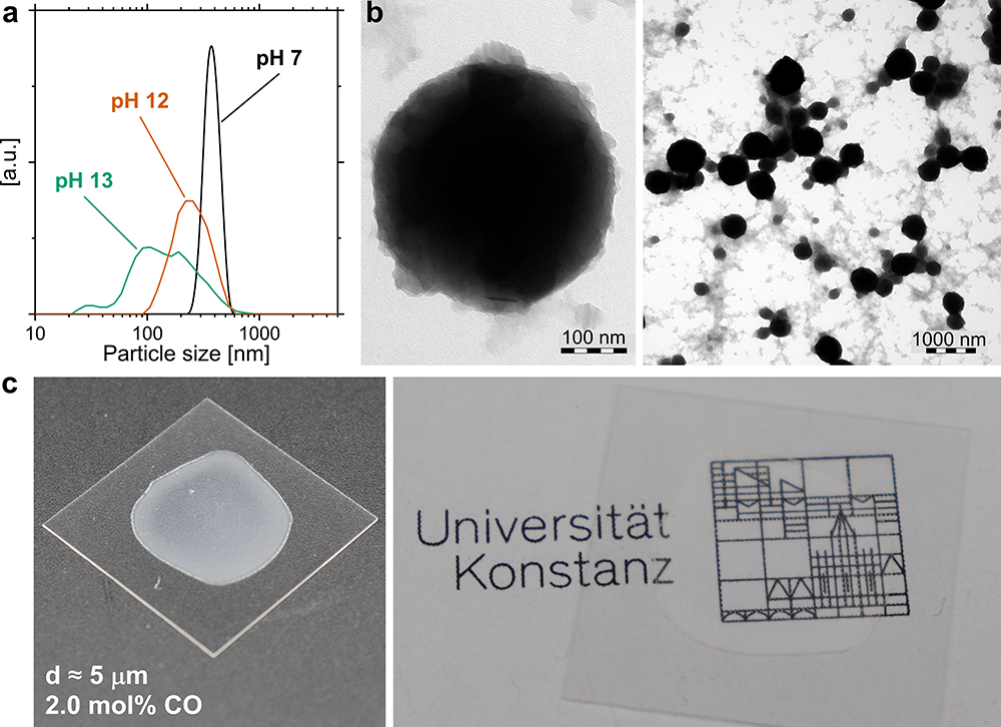
(a) Particle size distributions (volume averaged) determined by dynamic light scattering of aqueous keto-PE particle dispersions as obtained with **1-NH**_**2**_**PEG** at different pH (Table 1, entries 3, 6 and 7). (b) Transmission electron microscopy of keto-PE particles obtained with **1-NH**_**2**_**PEG** at pH 7 (Table 1, entry 3). (c) Keto-PE film (*d* ≈ 5 μm), formed by drop-casting from a dialyzed aqueous keto-PE dispersion (Table S2, entry 15).

In summary, we demonstrated the catalytic copolymerization of ethylene with carbon monoxide as a fundamental polar monomer in water to yield stable aqueous dispersions of in-chain keto-functionalized HDPE particles with high molecular weights. These can be accessed via nonalternating ethylene/CO copolymerization in water, which is enabled by advanced water-soluble [P,O]Ni(II) phosphinophenolate catalysts bearing either a hydrophilic labile NH_2_-PEG ligand or a hydrophilic −SO_3_Na substituent. The different pH dependence of the catalytic activity and observed polymer end groups suggest that additional termination mechanisms are involved compared to aqueous ethylene homopolymerizations. These termination reactions limit the catalyst productivity. A decreased productivity has also been observed for aqueous ethylene-acrylate copolymerizations in comparison to either aqueous ethylene homopolymerizations or nonaqueous acrylate copolymerizations by Ni(II) phosphinephenolate catalysts.^42^ We speculate that also here deactivation pathways specific to the aqueous environment occur from the polymerization intermediates formed by polar monomer enchainment, in this case hydrolysis of electron poor Ni-alkyls formed from acrylate insertion.^43^ Jian et al. have recently demonstrated how this issue can be addressed by the concept of methylene spacers between the functional group and the reacting double bond^44,45^ in aqueous polymerizations, enabling 7-fold higher productivity than observed by us for in-chain CO incorporation.^46^ On the other hand, the more costly functionalized 1-olefin comonomers required are converted only to a low amount due to the high chemoselectivity of neutral Ni(II) catalysts for ethylene over 1-olefins.^47^ This motivates further efforts toward more robust catalysts for the aqueous copolymerization of fundamental monomers in conjunction with appropriate reaction conditions.^48^ Our insights reported here reveal the feasibility of the approach, as well as the origin of current limitations, and can provide guidelines for further developments.

## Supporting Information Available

Syntheses and characterizations of precatalysts; Polymerization procedure; Polymer characterization data (IR spectra, NMR spectra, DSC traces, SEC data); Particle analysis of keto-PE dispersions (DLS, TEM) (PDF)
