# Supplementary material for: Aqueous Keto-Polyethylene Dispersions from Catalytic Copolymerization of Ethylene and Carbon Monoxide in Water
Source: ACS Macro Lett. 2024 Jun 24;13(7):841–6. doi: 10.1021/acsmacrolett.4c00313 (PMC11256749; doi:10.1021/acsmacrolett.4c00313)
Supplement: Supplementary file 1 — mz4c00313_si_001.pdf [file mz4c00313_si_001.pdf]

Supporting Information

for

Aqueous keto-polyethylene dispersion from catalytic copolymerization of ethylene and carbon monoxide in water

Maximilian Baur, Rosa Habè and Stefan Mecking*

Chair of Chemical Materials Science, Department of Chemistry, University of Konstanz, 78464 Konstanz, Germany.

E-mail: stefan.mecking@uni-konstanz.de

Table of Contents

I. Materials and Methods 3

Materials and general methods 3

Instruments and characterization 3

General procedure for aqueous polymerizations 4

Synthetic procedures 5

II. Supplementary experimental and characterization data 13

Complete copolymerization data 13

Aqueous ethylene homopolymerizations 15

Supplementary IR spectra of polymers 16

Supplementary NMR data of polymers 21

SEC traces of polymers 28

Thermal properties from differential scanning calorimetry 30

Dynamic light scattering (DLS) of keto-PE dispersions 31

Transmission electron microscopy (TEM) of keto-PE particles 32

Keto-PE film formation from aqueous particle dispersions 33

III. Supporting References 34

I. Materials and Methods

Materials and general methods

Unless noted otherwise, all manipulations of air and moisture sensitive materials were carried out under an inert gas atmosphere using standard glovebox and Schlenk techniques.
Solvents were dried and degassed using standard laboratory techniques. Benzene was distilled from sodium, THF from sodium benzophenone ketyl. Pentane and toluene were dried over molecular sieves (3 Å) and degassed by passing through a MB-SPS-800 solvent purification system by MBRAUN. Deionized water for aqueous polymerizations was distilled under a constant stream of N_2 to remove dissolved oxygen. Nickel precursor [(tmeda)NiMe$_2$] was prepared according to literature procedures.[1] 2-*tert*-butyl-4-fluoro phenol[2], 2-(4-fluoro phenoxy)tetrahydro-2*H*-pyran[3] and 2-(2-(*tert*-butyl)-4-fluorophenoxy)tetrahydro-2H-pyran were prepared according to reported procedures.[3] *α*-amino-ω-methoxy-polyethylene glycol (M_n 5516 g mol^{-1}) was purchased from Iris Biotech. 4-Fluoro phenol and sodium dodecyl sulfate (SDS) were purchased from Sigma Aldrich, and CsOH was supplied by ABCR. Ethylene of grade 3.5 and carbon monoxide of grade 4.7 were purchased from Air Liquide and used as received. Deuterated solvents were bought from Eurisotop. All other commercially available reagents were supplied by Sigma Aldrich, Acros, ABCR, or Activate Scientific.

Instruments and characterization

NMR-spectra were recorded on a Bruker Avance III 400, a Bruker Avance III HD or a JEOL ECZ 500R spectrometer. ^{1}H chemical shifts were referenced to the solvent residual proton signals (C_6D_6: 7.16 ppm, CD_3OD: 3.31 ppm, $C_2D_2Cl_4$: 6.00 ppm) and ^{13}C chemical shifts were referenced to the carbon signals of the deuterated solvent (C_6D_6: 128.1 ppm, CD_3OD: 49.0 ppm, $C_2D_2Cl_4$: 73.8 ppm). ^{19}F chemical shifts were referenced to external $BF_3 \cdot OEt_2$. Data evaluation was performed with MestreNova software by MestreLab SL. NMR spectra of polymers were acquired at 110 °C in 1,1,2,2-tertachlorethane-*d2*.
Size exclusion chromatography (SEC) was performed on a PolymerChar GPC-IR instrument equipped with an integrated four-capillary viscometer and an IR5 dual wavelength infrared detector (selective for methylene and methyl groups) on PSS Polefin Linear XL columns (3 × 30 cm) and with an additional guard column at 160 °C and 0.5 mL min^{-1} in 1,2-dichlorobenzene. Universal calibration using narrow polystyrene standards was employed. The raw data was evaluated with PSS WinGPC UniChrom software.

Differential scanning calorimetry (DSC) was performed on a Netzsch DSC 204 F1 with a bicyclic temperature program and heating/cooling rates of 10 K min^{-1}. For the measurements, the polymers were weighed into sealed 40 µL aluminum pans.
ATR-IR spectra of polymers were acquired on a Perkin Elmer Spectrum 100 instrument. Quantitative analysis of keto-contents was performed according to previously reported procedures.[4] For calculation of the carbon monoxide incorporation, the ratio of the intensity of the C=O signal (peak ~ 1714 cm^{-1}) to the intensity of the C-H signal of the PE at 2915 cm^{-1} was calculated and referenced with linear polyketone samples with known C=O content synthesized via ADMET copolymerization and subsequent hydrogenation (Figure S1).[5]

Dynamic Light Scattering (DLS) measurement was performed on a diluted polyethylene dispersion using a Malvern Zetasizer Nano-ZS ZEN 3600 instrument (633 nm) in backscattering mode (173°) at 25 °C. Particle size distribution was analyzed using the Malvern Zetasizer Software, version 7.12.
Transmission electron microscopy (TEM) images were acquired on a Zeiss Libra 120 EF-TEM instrument (120 kV). The respective samples were diluted to a solids content of ca. 0.03 wt. % and dialyzed in a Spectrum Laboratories Spectra/Por Dialysis Membrane 1, MWCO 6000-8000 with deionized water for 14 days to remove free sodium dodecyl sulfate. The resulting dispersions were dropped onto a TEM copper grid and dried for 2 h.
Keto-PE films were generated by dropcasting of copolymer dispersions (ca. 0.05 to 0.5 wt.% polymer content) on a cleaned glass surface. Particle dispersions were first dialyzed to remove excessive SDS, which could disturb film formation.

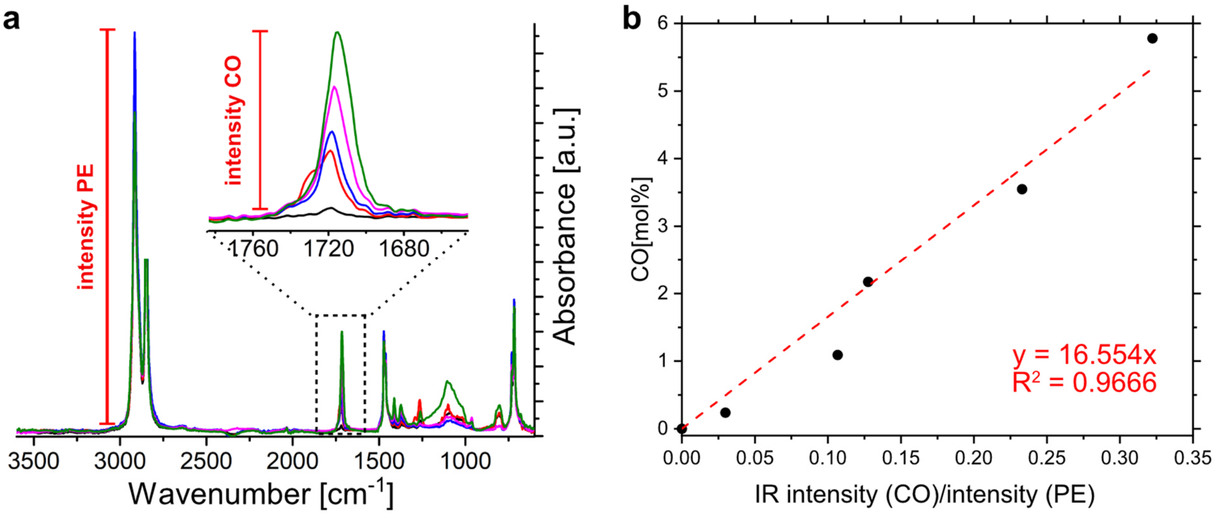

Figure S1. Referencing of IR spectra. **a:** Polyketones with known carbonyl contents were analyzed by ATR-IR. The samples were synthesized via ADMET copolymerization of docosa-1,21-dien-11-one and undeca-1,10-diene followed by hydrogenation.[5] The signal intensity used for calculation of ratios for carbonyl stretching vibrations (~ 1714 cm^{-1}) vs. the polyethylene C-H vibration (2915 cm^{-1}) are depicted in red. **b:** The intensity ratio is directly proportional to the concentration of C=O groups in the polymer χ. That is, $\chi \approx n_{CO}/n_{C2H4}$ which is a valid approximation if $n_{C2H4} >> n_{CO}$.

General procedure for aqueous polymerizations

All polymerization experiments were carried out in a high-pressure polymerization set-up consisting of a 300 mL MiniClave by BüchiGlasUster equipped with a Cyclone 075 magnetically coupled mechanical pitched blade stirrer, in- and outlet valves, a temperature sensor, a digital pressure sensor, a Julabo CF41 thermostat and continuous (co-)monomer gas feeds. Gas flows of ethylene and carbon monoxide were individually monitored and regulated by El-Flow mass flow controllers by Bronkhorst. Gas feeds and temperature were monitored and controlled *via* LabVision software automatization by Hitec Zang.
The reactor was evacuated and purged with nitrogen three times prior to the reaction while the internal temperature was > 70 °C. Sodium dodecyl sulfate (SDS) and CsOH were dissolved in 100 mL of deionized and deoxygenized water. For polymerizations which required low amounts of CsOH (pH 9.8 and 10.8) a respective stock solution was used instead of solid CsOH. For polymerizations under acidic conditions, $KHSO_4$ was used to adjust the desired pH value instead of CsOH. 90 mL of the obtained SDS/CsOH solution were transferred to the reactor vessel by cannula while 10 mL were used to dissolve the respective precatalysts. After heating to the desired reaction temperature, the precatalyst solution was added to the reactor by cannula transfer. The stirring rate was adjusted to 1000 rpm and the reactor was pressurized to 30 bar with the desired mixture of carbon monoxide and ethylene. This mixture was further fed to the reactor by the automated mass flow controllers to maintain a stable pressure (thus replenishing consumed gaseous monomer) over the course of the entire reaction. After the desired polymerization time, the reactor was vented and cooled. The obtained polymer dispersion was weighed and separated in two parts: 10-50 wt.% were filtered over cotton wool and used for dispersion and particle analysis. The remaining portion was precipitated in methanol (1000 mL). The precipitated polymers were filtered off, washed thoroughly with water and methanol and vacuum dried at 60 °C for > 24 h. Reference homopolymerizations of ethylene were carried out following the same protocol, without addition of CO to the gas feed.

Synthetic procedures

Synthesis of phosphinophenols

Phosphinophenols **[P,O]-1**, **[P,O]-2**, **[P,O]^F-3c, [P,O]^F-3d, [P,O]^F-4c** and **[P,O]^F-4d** were prepared according to reported procedures.[7–10]

General procedure for the synthesis of hydrophilic catalyst precursors

The *α*-amino-PEG complexes were synthesized according to a procedure reported for salicylaldiminato complexes.[11] A solution of the respective phosphinophenol (1.05 equiv.) in benzene (4 mL) was added to [(tmeda)NiMe$_2$] (1.10 equiv.). During the addition, methane evolution was observed and the resulting orange solution was stirred at r.t. for 1 h. A solution of NH_2-PEG-OMe (M= 5516 g mol^{-1}, 1.00 equiv.) in 1 mL of benzene was added and the reaction was stirred for further 3 h at r.t.. The resulting orange solution was filtered *via* syringe filter to remove nickel black. The clear solution was vitrified by cooling the flask in liquid nitrogen and volatile compounds were removed by sublimation in vacuum. The obtained solid was washed with pentane (3 x 5 mL) and dried in vacuum to give the respective complex as yellow solid.
Complexes **3^{SO3}-pyr** and **4^{SO3}-pyr** with pyridine as labile ligand were synthesized according to a reported procedure.[10]

Synthesis of complex 1-NH$_2$PEG:

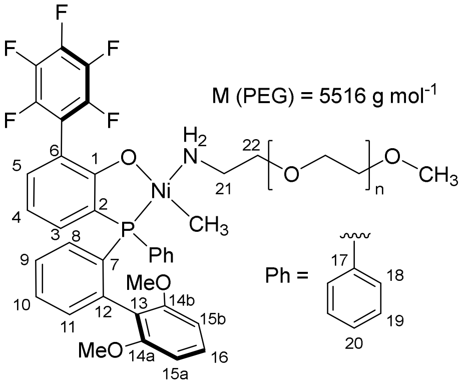

Complex **1-NH$_2$PEG** was prepared following the general procedure, using 61.0 mg of ligand **[P,O]-1** (105 µmol, 1.05 equiv.), 22.5 mg of [(tmeda)NiMe$_2$] (110 µmol, 1.10 equiv.) and 551.6 mg of NH_2-PEG-OMe (100 µmol, 1.00 equiv.) in 5 mL of benzene. Yield: 563.4 mg, 91 µmol, 87 %.

^{1}H NMR (400 MHz, C_6D_6, 300 K): δ [ppm] = 7.57 – 7.44 (m, 3H, H-9, H-11, -Ph), 7.41 (t, J = 6.1 Hz, 1H, H-10), 7.32 (t, J = 8.4 Hz, 1H, H-8)), 7.21 (t, J = 8.1 Hz, 3H, H-5, H-16, -Ph), 7.02 - 6.97 (4H, H-3, H-18, H-19), 6.53 (t, J = 7.3 Hz, H-20), 6.42 (d, J = 8.4, 4.3 Hz, 2H, H-15a/ b), 3.69 – 3.39 (m, 500H, *H*-PEG), 3.37 (3H, -OMe^b)* 3.13 (s, 5H, PEG-OC*H*$_3$ + H-22), 3.04 (s, 3H, -OMe^a)*, 2.37 (s, 2H, H-21), 1.47 (s, 2H, -N*H*$_2$), -1.25 (d, $^3J_{HP}$ = 5.3 Hz, 1H, Ni-C*H*$_3$).
* overlapping with *H*-PEG signal.

^{31}P NMR (162 MHz, C_6D_6, 300K): δ [ppm] = 21.07

^{19}F NMR (376 MHz. C_6D_6, 300 K): δ [ppm] = - 136.59 (dd, J = 24.2, 7.7 Hz, *o*-C*F*), - 142.15 (dd, J = 24.7, 7.9 Hz, *o*-C*F*), - 159.56 (t, J = 21.4 Hz, *p*-C*F*), - 164.68 (td, J = 23.3, 8.0 Hz, *m*-C*F*), - 165.53 (td, J = 22.5, 7.7 Hz, *m*-C*F*).

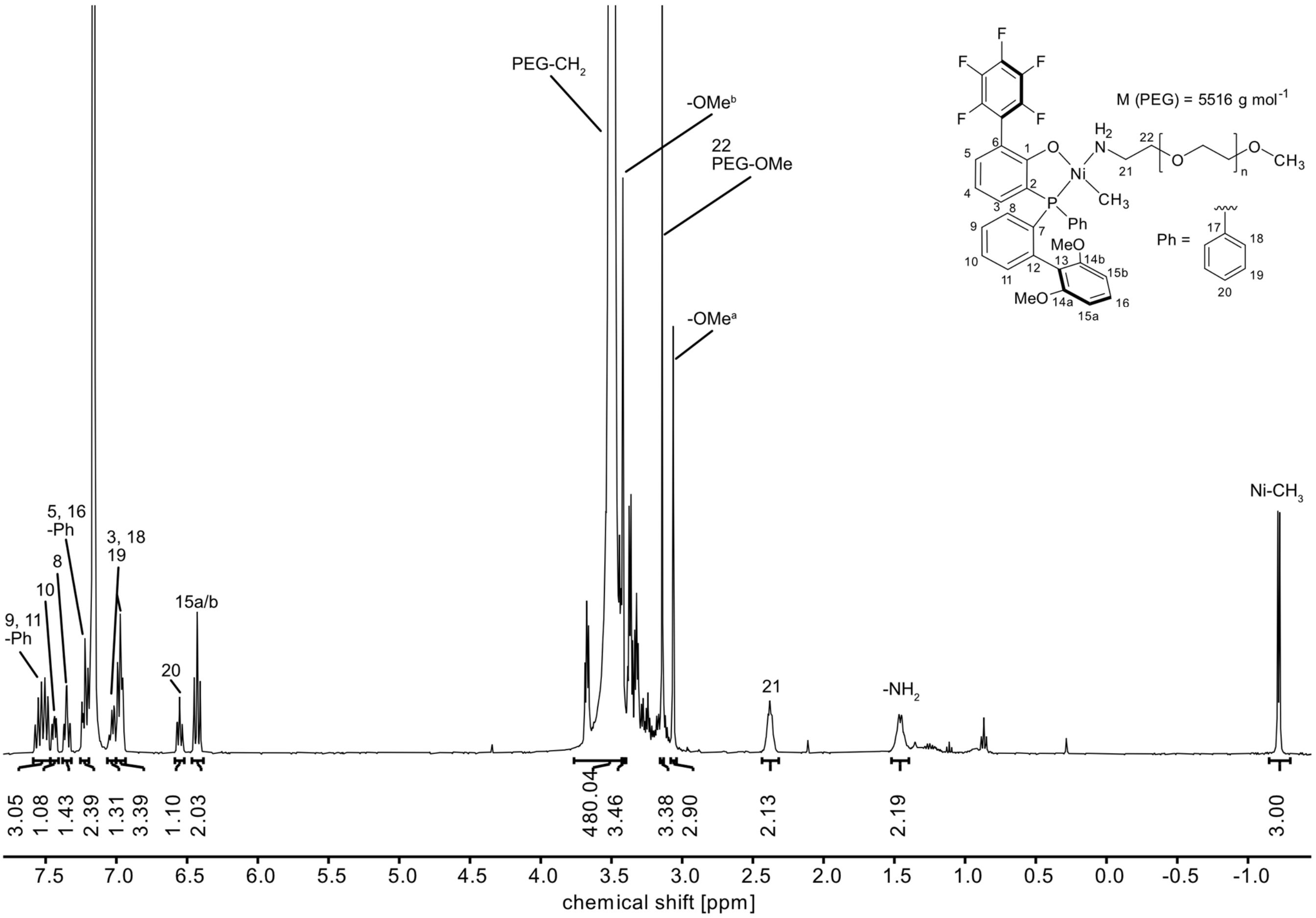

Figure S2. ^{1}H NMR spectrum (400 MHz, 300K, C_6D_6) of **1-NH₂PEG**.

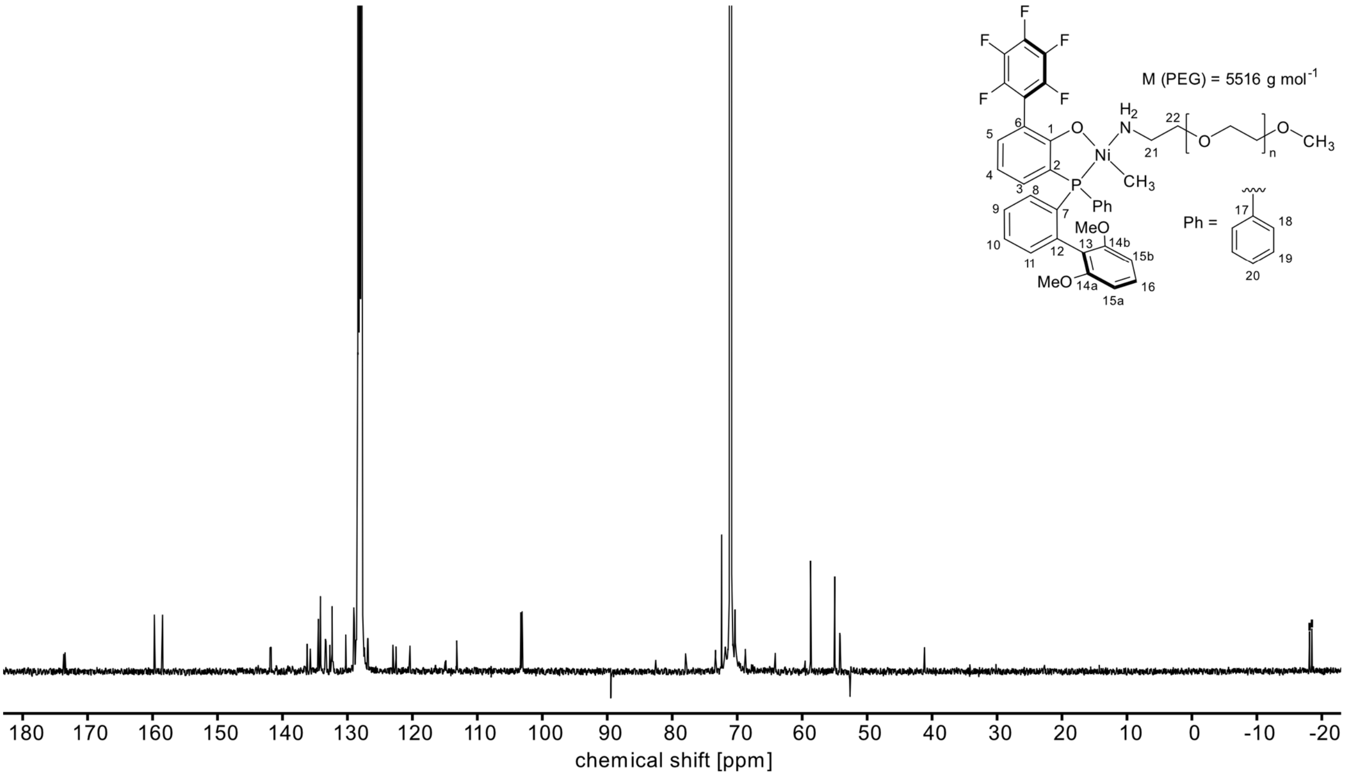

Figure S3. ^{13}C NMR spectrum (101 MHz, 300K, C_6D_6) of **1-NH₂PEG**.

Synthesis of complex 2-NH$_2$PEG:

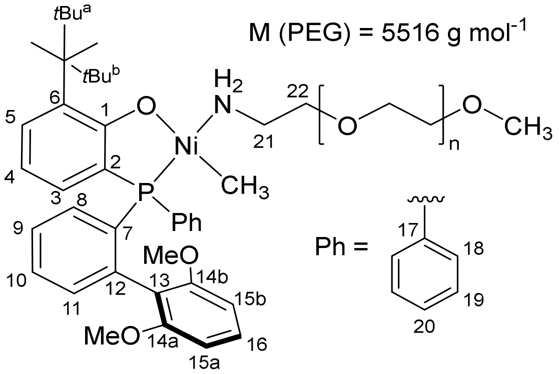

Complex **2-NH$_2$PEG** was prepared following the general procedure, using 49.4 mg of ligand **[P,O]-2** (105 µmol, 1.05 equiv.), 22.5 mg of [(tmeda)NiMe$_2$] (110 µmol, 1.10 equiv.) and 551.6 mg of NH$_2$-PEG-OMe (100 µmol, 1.00 equiv.) in 5 mL of benzene. The product was washed another three times with pentane to give pure **2-NH$_2$PEG**. Yield: 545.1 mg, 90 µmol, 90 %.

^{1}H NMR (400 MHz, C_6D_6, 300 K): δ [ppm] = 7.56 – 7.40 (m, 4H, H-9, H-10, -Ph), 7.31 – 7.18 (m, 4H, H-3 + H-5 + H-8 + H-16), 7.04 – 6.91 (m, 4H, -Ph), 6.58 – 6.42 (m, 3H, H-4, H-15a/b), 3.71 – 3.25 (m, 700H, PEG-C*H*$_2$), 3.45 (s, 3H, -OMe^b)*, 3.12 (m, 5H, H-22 + PEG-OC*H*$_3$), 2.89 (s, 3H, -MeO^a), 2.54 (brs, 2H, H-21), 1.69 (brs, 2H, -N*H*$_2$), 1.58 (s, 9H, *t*Bu), – 1.16 (d, *J* = 4.9 Hz, 3H, Ni-C*H*$_3$).
* overlapping with *H*-PEG signal.

^{13}C NMR (101 MHz, C_6D_6, 300K): δ [ppm] = 175.0 (d, *J* = 21.0 Hz, C-1), 159.9 (C-14a), 158.4 (C-14b), 141.7 (d, *J* = 17.4 Hz, C-12), 137.3 (C-6), 137.0 (d, *J* = 40.2 Hz, C-17), 135.0 (C-3,4,9,10 or -Ph), 133.2 (d, *J* = 9.3 Hz, C-3,4,9,10 or -Ph), 132.9 (d, *J* = 49.9 Hz, C-7), 132.3 (d, *J* = 9.4 Hz, C-3,4,9,10 or -Ph), 130.5 (C-16), 129.9 (d, J = 2.0 Hz, C-3 or C-5), 129.0 (C-3,4,9,10 or -Ph), 128.7 (-Ph), 128.6 (d, *J* = 2.0 Hz, -Ph), 126.6 (d, *J* = 6.6 Hz, C-3,4,9,10 or -Ph), 120.4 (d, *J* = 5.4 Hz, C-13)*, 120.3 (d, J = 50.3 Hz, C-2)*, 113.3 (d, *J* = 7.7 Hz, C-4), 103.2 (C-15a or b), 103.0 (C-15a or b), 72.4 and 72.0 – 70.0 (m, C-PEG), 58.7 (PEG-OCH$_3$), 55.0 (-OMe^b), 54.1 (-OMe^a), 35.2 (*t*Bu^b), 29.9 (*t*Bu^a), -18.6 (d, *J* = 34.9 Hz, Ni-*C*H$_3$).
* overlapping signals

^{31}P NMR (162 MHz, C_6D_6, 300K): δ [ppm] = 21.39.

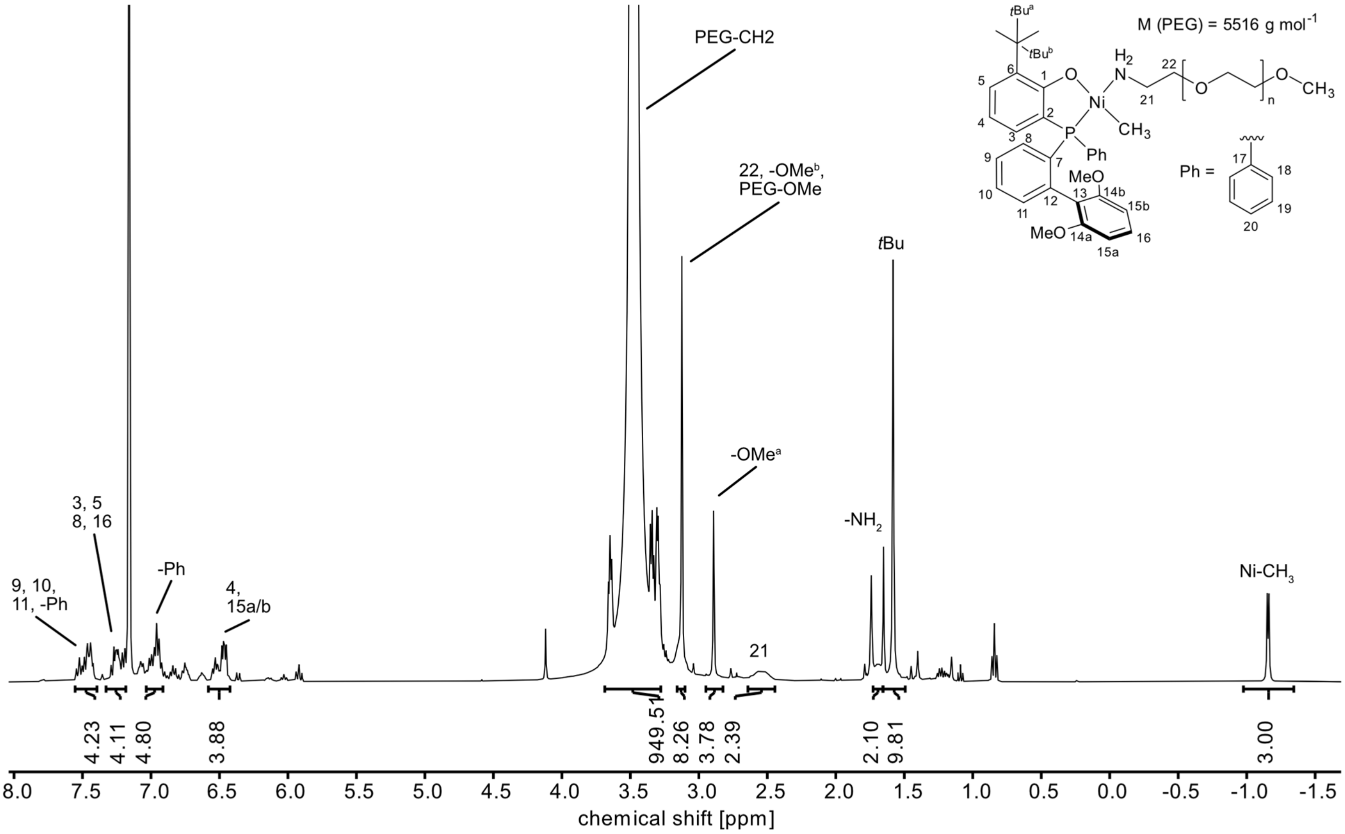

Figure S4. ^{1}H NMR spectrum (400 MHz, 300K, C_6D_6) of **2-NH2PEG**.

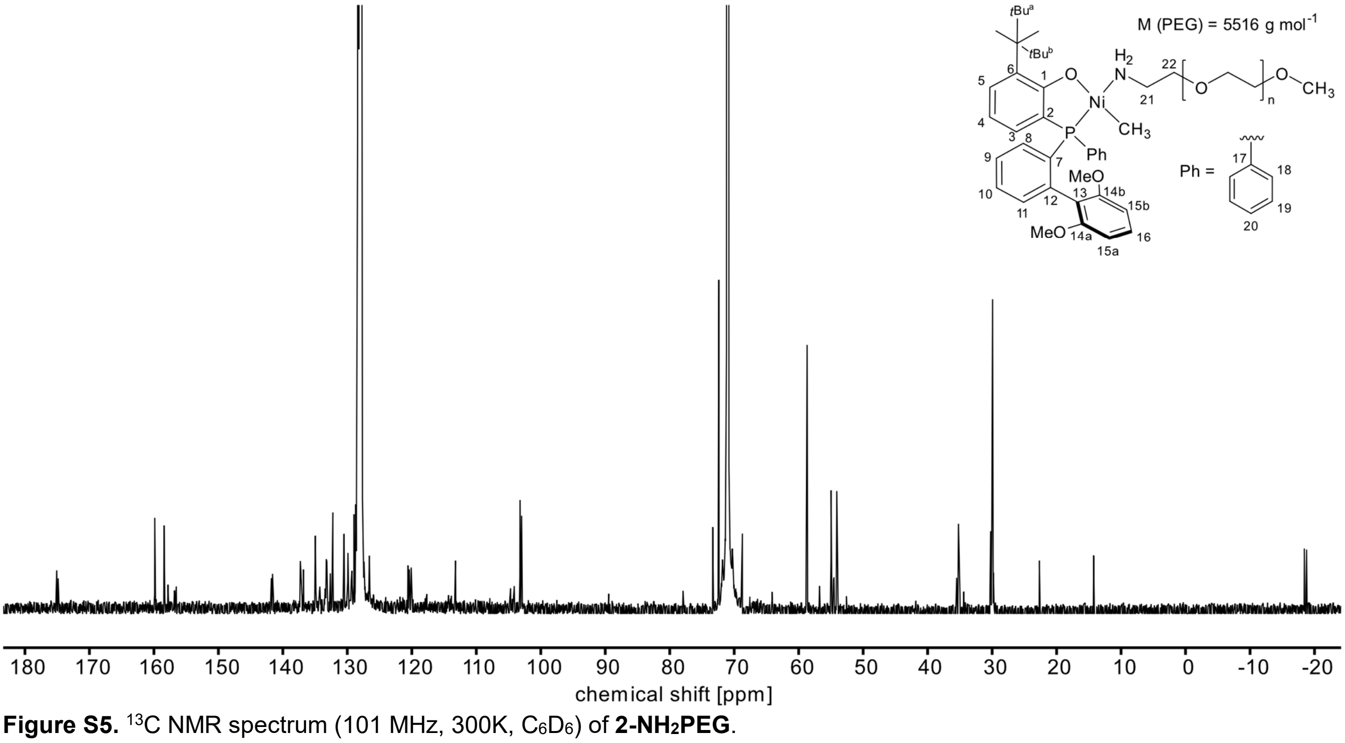

Figure S5. ^{13}C NMR spectrum (101 MHz, 300K, C_6D_6) of **2-NH2PEG**.

Synthesis of complex 3-NH$_2$PEG:

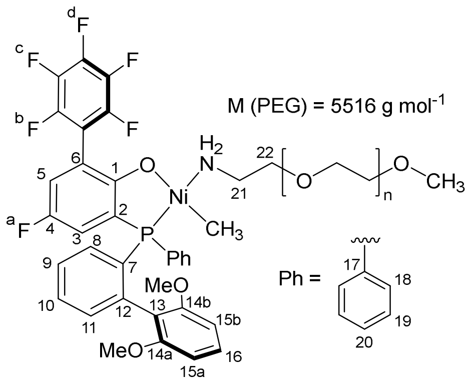

Complex **3-NH$_2$PEG** was prepared following the general procedure, using 62.8 mg of ligand **[P,O]^F-3c** (105 µmol, 1.05 equiv.), 22.5 mg of [(tmeda)NiMe$_2$] (110 µmol, 1.10 equiv.) and 551.6 mg of NH$_2$-PEG-OMe (100 µmol, 1.00 equiv.) in 5 mL of benzene. Yield: 543.2 mg, 88 µmol, 88 %.

^{1}H NMR (400 MHz, C_6D_6, 300 K): δ [ppm] = 7.51 – 7.44 (m, 2H, H-9 + H-11), 7.43 – 7.34 (m, 2H, H-8 + H-10), 7.25 – 7.19 (m, 2H, H-5 + H-16)*, 7.07 – 6.93 (m, 4H, H-3, H-18, H-19), 6.90 (t, *J* = 7.6 Hz, 1H, H-20), 6.41 (d, *J* = 8.4 Hz, 2H, H-15a/b) 3.74 – 3.25 (m, 500H, H-PEG), 3.41 (s, 3H, -OMe^b)**, 3.13 (s, 5H, H-22 + PEG-OC*H*$_3$), 3.06 (s, 3H, -OMe^a), 2.35 (s, 2H, H-21), 1.46 (s, 2H, -N*H*$_2$), -1.25 (d, *J* = 5.4 Hz, 3H, Ni-C*H*$_3$).
* overlapping with solvent signal, ** overlapping with *H*-PEG signal.

^{13}C NMR (101 MHz, C_6D_6, 300K): δ [ppm] = 170.3 (d, *J* = 21.6 Hz, C-1), 159.6 (C-14a), 158.4 (C-14b), 152.3 (d, *J* = 231.6 Hz, C-4), 141.8 (d, *J* = 17.9 Hz, C-12), 135.3 (d, *J* = 49.2 Hz, C-7), 134.3 (C-8), 133.3 (d, *J* = 9.5 Hz, C-9 or C-11), 132.3 (d, *J* = 9.7 Hz, C-9 or C-11), 131.8 (d, *J* = 49.8 Hz, C-17), 130.5 (C-10), 129.11 (C-16),127.4 (C-20), 127.1 (d, *J* = 6.3 Hz, C-18 or C-19), 123.0 (d, *J* = 45.9 Hz, C-2), 120.9 (d, *J* = 24.7 Hz, C-3), 120.3 (d, J = 5.6 Hz, C-13), 119.1 (d, *J* = 21.6 Hz, C-5) 103.3 (C-15a/b), 103.2 (C-15a/b), 72.4 and 72.1 – 69.9 (m, C-PEG), 58.7 (PEG-OCH$_3$), 55.0 (-OMe^b), 54.3 (-OMe^a), -18.18 (d, *J* = 35.5 Hz, Ni-*C*H$_3$).
* overlapping with solvent signal

^{31}P NMR (162 MHz, C_6D_6, 300K): δ [ppm] = 21.02.

^{19}F NMR (376 MHz, C_6D_6, 300 K) δ [ppm] = -133.23 (d, *J* = 3.3 Hz, 1F, ^{a}F), -136.60 (dd, *J* = 24.2, 7.8 Hz, 1F, ^{b}F), -141.74 (dd, *J* = 24.4, 7.8 Hz, 1F, ^{b}F), -158.69 (t, *J* = 21.6 Hz, 1F, ^{d}F), -164.34 (td, *J* = 22.8, 7.6 Hz, 1F, ^{c}F), -165.20 (td, *J* = 22.9, 7.7 Hz, 1F, ^{c}F).

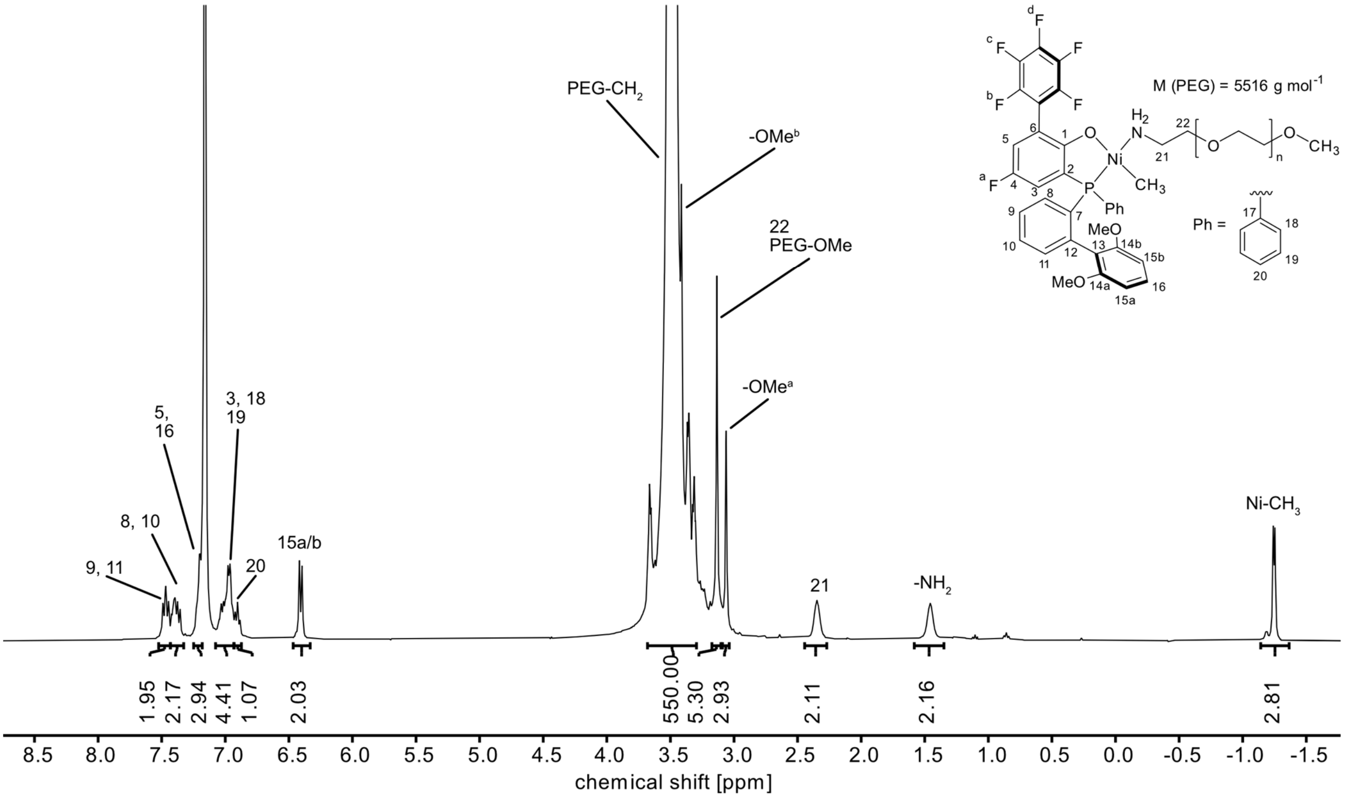

Figure S6. ^{1}H NMR spectrum (400 MHz, 300K, C_6D_6) of **3-NH₂PEG**.

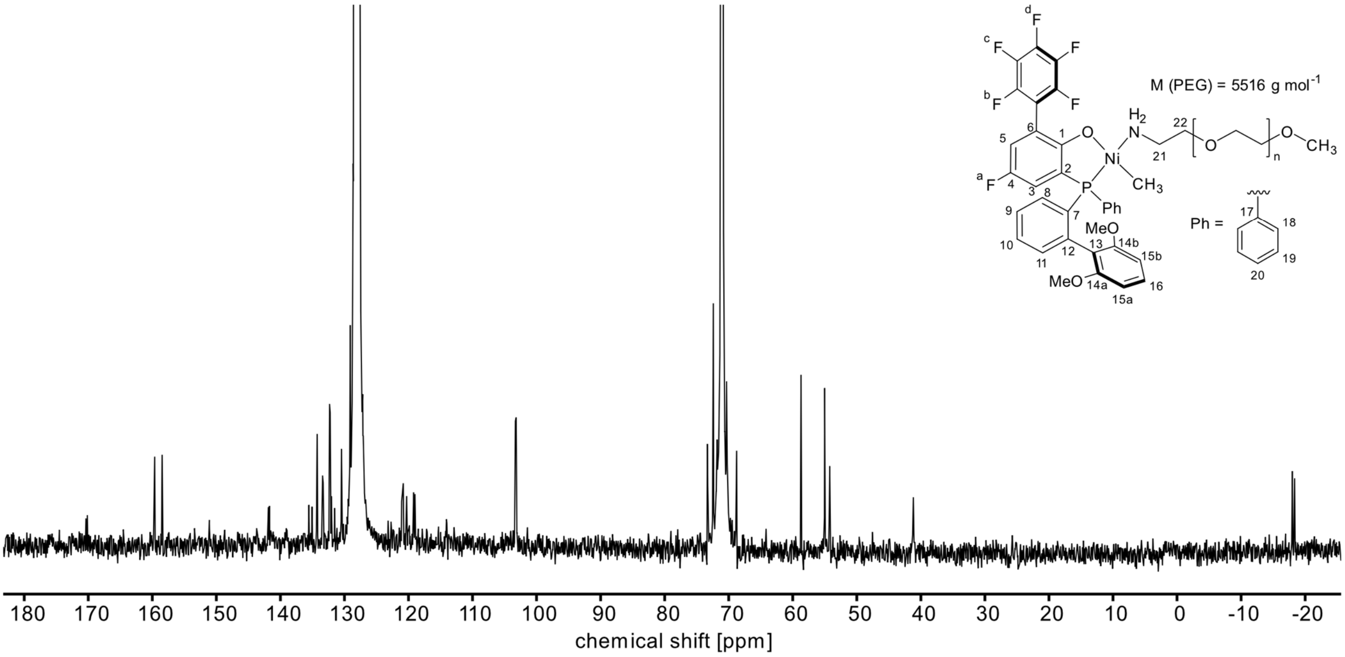

Figure S7. ^{13}C NMR spectrum (101 MHz, 300K, C_6D_6) of **3-NH₂PEG**.

Synthesis of complex 4-NH_2PEG:

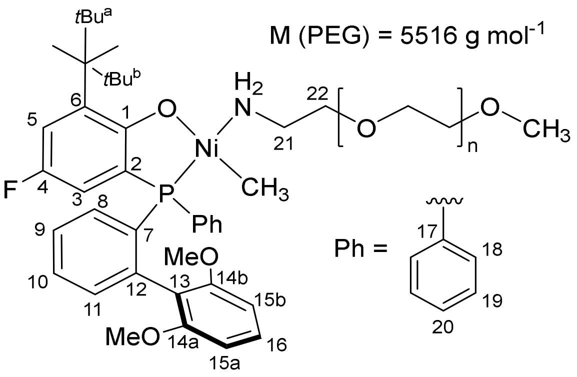

Complex **4-NH_2PEG** was prepared following the general procedure, using 51.3 mg of ligand **[P,O]^F-4c** (105 µmol, 1.05 equiv.), 22.5 mg of [(tmeda)$NiMe_2$] (110 µmol, 1.10 equiv.) and 551.6 mg of NH_2-PEG-OMe (100 µmol, 1.00 equiv.) in 5 mL of benzene. Yield: 569.3 mg, 94 µmol, 94 %.

^{1}H NMR (400 MHz, C_6D_6, 300 K): δ [ppm] = 7.48 – 7.34 (m, 4H, H-8 + H-9 + H-18 or H-19), 7.25 (t, *J* = 8.3 Hz, 1H, H-16)**, 7.22 – 7.16 (m, 1H, H-10)**, 7.11 (dd, *J* = 10.8, 3.1 Hz, 1H, H-5), 7.03 – 6.93 (m, 4H, H-3 + H-18 or H-19 + H-20), 6.89 (t, *J* = 7.5 Hz, 1H, H-11), 6.46 (d, *J* = 8.3 Hz, 1H, H-15b), 6.42 (d, *J* = 8.3 Hz, 1H, H-15a), 3.74 – 3.25 (m, 500H, *H*-PEG), 3.46 (s, 3 H, -MeO^b)*, 3.12 (m, 5H, H-22 + PEG-OCH_3), 2.92 (s, 3H, -MeO^a), 2.52 (brs, 2H, H-21), 1.67 (brs, 2H, -NH_2) 1.47 (s, 9H, *t*Bu), – 1.18 (d, *J* = 5.0 Hz, 3H, Ni-CH_3).
* overlapping with *H*-PEG signal, ** overlapping signals

^{13}C NMR (101 MHz, C_6D_6, 300K): δ [ppm] = 171.4 (d, *J* = 20.7 Hz, C-1), 159.7 (C-14a), 158.4 (C-14b), 153.1 (dd, *J* = 227.0, 9.8 Hz, C-4), 141.6 (d, *J* = 17.3 Hz, C-12), 137.9 (dd, *J* = 9.7, 5.3 Hz, C-6), 136.4 (d, *J* = 47.2 Hz, C-17), 134.70 (d, *J* = 1.8 Hz, C-8), 133.2 (d, *J* = 9.2 Hz, C-Ph), 132.2 (d, *J* = 49.7 Hz, C-7)*, 132.2 (d, *J* = 9.6 Hz, C-9)*, 130.1 (d, *J* = 2.3 Hz, C-Ph), 129.1 (C-16), 128.8 (d, *J* = 2.2 Hz, C-Ph), 126.9 (d, *J* = 6.7 Hz, C-11), 120.2 (d, *J* = 5.3 Hz, C-13), 119.7 (dd, *J* = 49.7, 6.1 Hz, C-2), 116.7 (d, *J* = 24.3 Hz, C-5), 114.0 (d, *J* = 20.9 Hz, C-3), 103.2 (C-15a or b), 103.1 (C-15a or b), 72.4 and 72.2 – 69.9 (m, C-PEG + C-21 + C22), 58.7 (PEG-OCH_3), 55.0 (-OMe^b), 54.2 (-OMe^a), 35.3 (*t*Bu^b), 29.5 (*t*Bu^a), -18.5 (d, *J* = 35.2 Hz, Ni-CH_3).
* overlapping signals

^{31}P NMR (162 MHz, C_6D_6, 300K): δ [ppm] = 21.68.

^{19}F NMR (376 MHz, C_6D_6, 300K): δ [ppm] = – 132.95 (d, J_{FP} = 3.3 Hz).

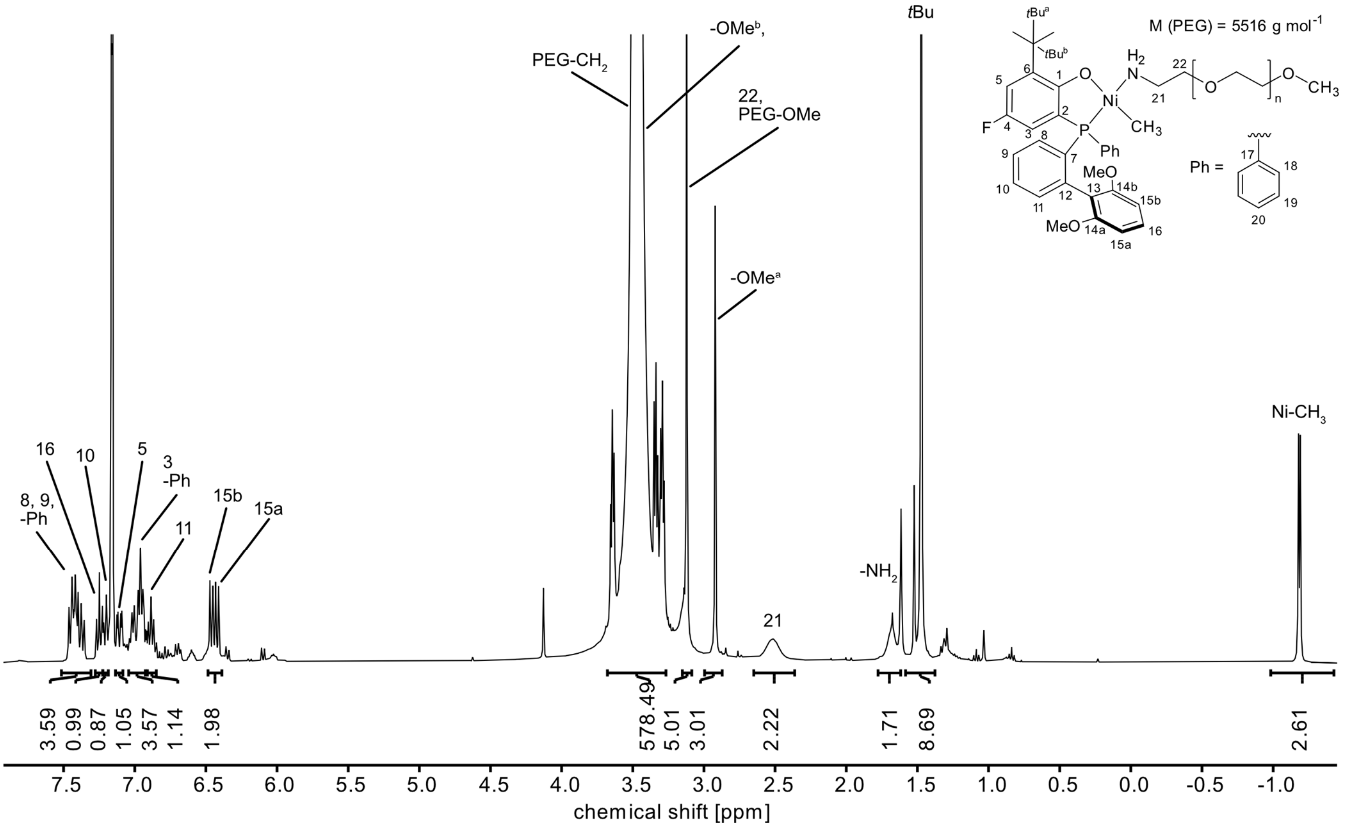

Figure S8. ^{1}H NMR spectrum (400 MHz, 300K, C_6D_6) of **4-NH₂PEG**.

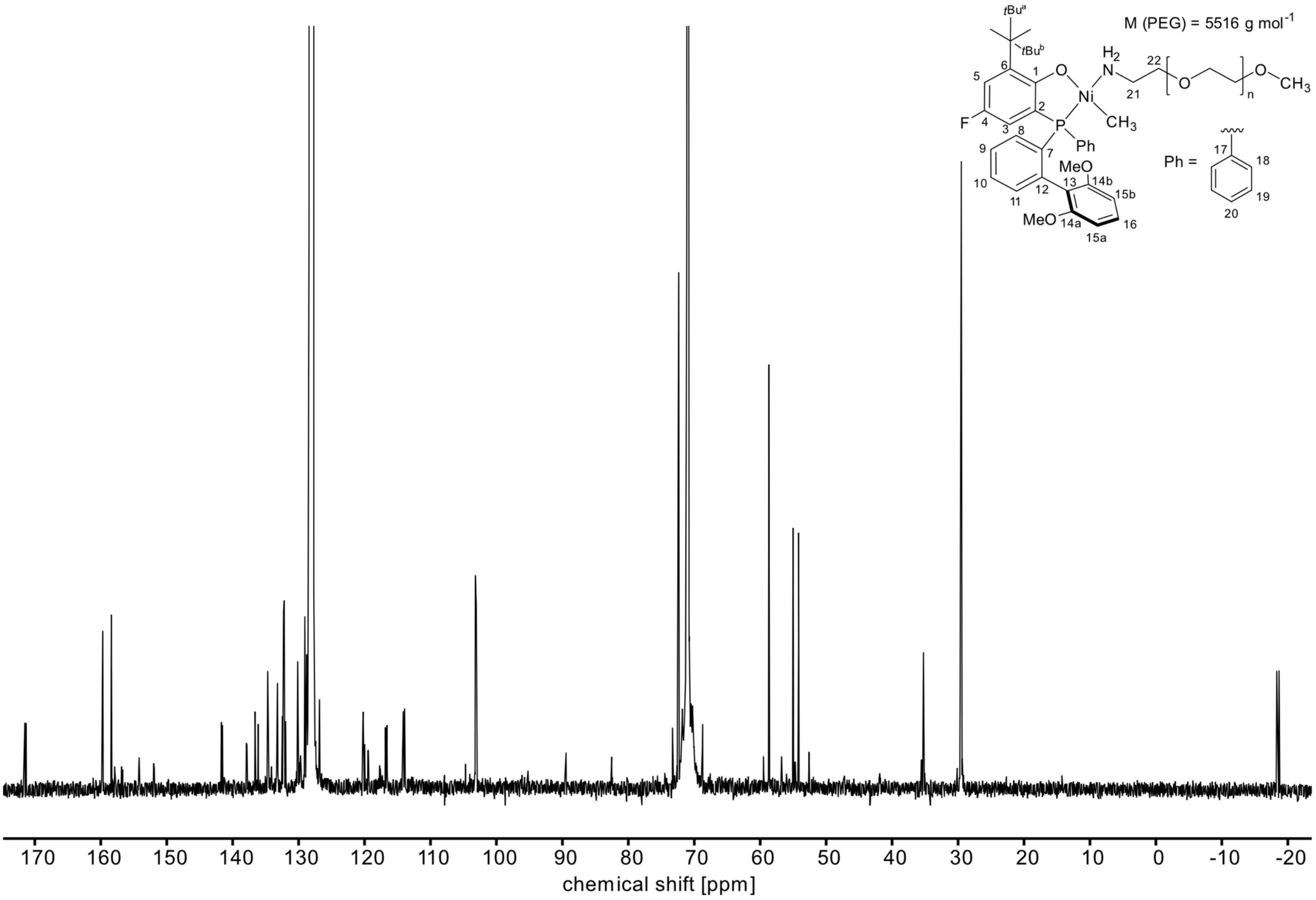

Figure S9. ^{1}H NMR spectrum (400 MHz, 300K, C_6D_6) of **4-NH₂PEG**.

II. Supplementary experimental and characterization data

Complete copolymerization data

Table S1. Results of catalytic ethylene/CO copolymerizations in aqueous reaction media at different ethylene-CO feed ratio.

#	Cat.	CO/C_2H_4 in feed [%]†	Yield [g]	X‡ [mol%]	TOF§	M_n [10^3 g mol^{-1}] (M_w/M_n)*	T_m [°C] (Cryst. %)**
1	**1-NH₂PEG**	0.6	2.03	-	7.26	76 (1.5)	136 (68)
2	**1-NH₂PEG**	1.0	0.81	0.1	2.88	91 (1.6)	135 (68)
3	**1-NH₂PEG**	1.2	0.10	0.9 (1.2)	0.36	27 (1.3)	133 (75)
4	**1-NH₂PEG**	1.4	0.02	1.3	0.06	13 (1.3)	n.d.
5	**1-NH₂PEG**	2.0	traces	n.d.	-	n.d.	n.d.

Polymerization conditions: 10 µmol precatalyst loading, 90 °C, 30 bar, 1 h, 100 mL of oxygen free water, pH 12.8 (1.0 g CsOH), 750 mg sodium dodecyl sulfate (SDS), 1000 rpm pitched blade stirrer. † Carbon monoxide content in an automated ethylene-CO gas feed. ‡ Carbon monoxide incorporation determined by IR spectroscopy. In brackets: Carbon monoxide incorporation determined from ^1H NMR spectroscopy. § TOF given in units of 10^3 mol [C_2H_4] mol^{-1} [Ni] h^{-1}. * Determined by SEC in 1,2-dichlorobenzene at 160 °C (0.5 mL min^{-1}) *via* universal calibration versus narrow polystyrene standards. ** Determined by DSC (10 K min^{-1}), second heating cycle.

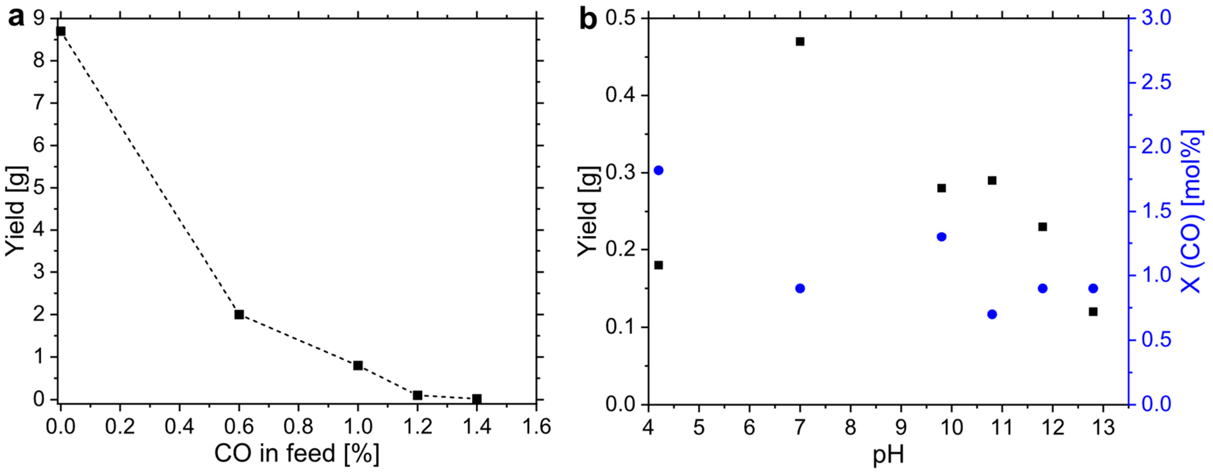

Figure S10. a: Inhibitory effect of the presence of CO in the monomer feed on copolymerization yield (Table S1). **b:** Comparison of C=O incorporation ratios (blue) and yields (black) in dependence of pH. Copolymers obtained from polymerization employing complex **1-NH₂PEG** (Table S2, entries 2 - 7).

Table S2. Results of aqueous ethylene/CO copolymerizations at different pH values of the reaction medium. Note that entries 1 – 7 are identical to Table 1 in the manuscript.

#	Cat.	pH	Yield [g]	X‡ [mol%]	TOF§	M_n (M_w/M_n)* [10^3 g mol^{-1}]	T_m [°C] (Cryst. %)**	Z-Avrg. [nm] ‡‡
1	**1-NH$_2$PEG**	2.2	no yield	-	-	-	-	-
2	**1-NH$_2$PEG**	4.2	0.18	1.8	0.6	68 (1.5)	135 (70)	653 ± 49
3	**1-NH$_2$PEG**	7	0.47	0.9	1.7	80 (1.6)	134 (71)	1008 ± 92
4	**1-NH$_2$PEG**	9.8	0.28	1.3	1.0	57 (1.6)	133 (71)	748 ± 55
5	**1-NH$_2$PEG**	10.8	0.29	0.7	1.0	47 (1.6)	134 (73)	331 ± 7
6	**1-NH$_2$PEG**	11.8	0.23	0.9	0.8	40 (1.6)	134 (73)	241 ± 2
7	**1-NH$_2$PEG**	12.8	0.14	0.7	0.5	29 (1.5)	132 (72)	147 ± 3
8	**2-NH$_2$PEG**	7	traces	n.d.	n.d.	n.d.	n.d.	n.d.
9	**2-NH$_2$PEG**	9.8	0.005	~ 14^x	n.d.	n.d.	n.d.	n.d.
10	**2-NH$_2$PEG**	10.8	0.02	~ 25^x	0.1	32 (2.6)	n.d.	362 ± 150
11	**2-NH$_2$PEG**	12.8	0.03	1.0	0.1	20 (1.5)	132 (73)	442 ± 119
12	**3-NH$_2$PEG**	7	0.21	2.0	0.8	47 (1.4)	134 (79)	563 ± 11
13	**3-NH$_2$PEG**	9.8	0.19	1.4	0.7	41 (1.4)	135 (72)	331 ± 3
14	**3-NH$_2$PEG**	10.8	0.06	1.7	0.2	24 (1.5)	132 (70)	272 ± 1
15	**3-NH$_2$PEG**	11.8	0.05	2.0	0.2	20 (1.5)	132 (71)	194 ± 3
16	**3-NH$_2$PEG**	12.8	0.06	0.9	0.2	19 (1.4)	132 (69)	212 ± 10
17	**4-NH$_2$PEG**	7	0.04	10	0.1	37 (1.8)	132 (45)	100 ± 1
18	**4-NH$_2$PEG**	9.8	0.05	9	0.1	41 (1.8)	133 (49)	164 ± 49
19	**4-NH$_2$PEG**	10.8	0.03	22^x	0.1	n.d.	122 (25)	392 ± 130
20	**4-NH$_2$PEG**	12.8	0.10	1.0	0.3	26 (1.5)	133 (73)	178 ± 4
21	**3^{SO3}-pyr**	7	0.24	2.4	0.9	16 (1.4)	129 (77)	210 ± 32
22	**3^{SO3}-pyr**	9.8	0.59	1.4	2.1	10 (1.6)	128 (76)	124 ± 7
23	**3^{SO3}-pyr**	10.8	0.31	1.9	1.1	12 (1.6)	130 (77)	121 ± 16
24	**3^{SO3}-pyr**	11.8	0.82	1.7	9.1	9 (2.0)	130 (78)	132 ± 34
25	**3^{SO3}-pyr**	12.8	0.10	0.7	0.4	3 (2.7)	126 (73)	287 ± 25
26	**4^{SO3}-pyr**	7	traces	n.d.	n.d.	n.d.	n.d.	n.d.
27	**4^{SO3}-pyr**	9.8	0.005	< 50^x	n.d.	n.d.	n.d.	n.d.
28	**4^{SO3}-pyr**	10.8	0.01	22^x	n.d.	22 (2.1)	n.d.	n.d.
29	**4^{SO3}-pyr**	11.8	0.16	2.0	0.6	41 (1.4)	134 (75)	82 ± 12
30	**4^{SO3}-pyr**	12.8	0.04	2.6	0.1	7 (2.3)	128 (71)	210 ± 45

*Polymerization conditions: 10 µmol precatalyst loading, 90 °C, 30 bar, 100 mL of oxygen free water, 750 mg sodium dodecyl sulfate (SDS), 1.2 % CO/C_2H_4 in an automated gas feed, 1000 rpm pitched blade stirrer, 60 min. ‡ Carbon monoxide incorporation determined by IR spectroscopy. § TOF given in units of 10^3 mol [C_2H_4] mol^{-1} [Ni] h^{-1}. * Determined by SEC in 1,2-dichlorobenzene at 160 °C, 0.5 mL min^{-1} via universal calibration versus narrow polystyrene standards. ** Determined by DSC (10 K min^{-1}), second heating cycle. ‡‡ Determined by dynamic light scattering. X Approximate CO content obtained from ATR-IR spectroscopy. Note that the employed calibration method is not accurate at very high CO incorporation ratios.

Aqueous ethylene homopolymerizations

Table S3. Results of ethylene homopolymerizations in aqueous media.

#	Cat.	pH	Yield [g]	TOF§	M_n [10^3 g mol^{-1}] (M_w/M_n)*	T_m [°C] (Cryst. %)**
1	**1-NH2PEG**	7	3.97	28.4	138 (1.6)	136 (66)
2	**1-NH2PEG**	10.8	5.61	40.1	130 (1.8)	136 (63)
3	**1-NH2PEG**	12.8	8.71	62.2	102 (1.6)	137 (64)
4	**2-NH2PEG**	7	0.11	0.8	166 (1.9)	137 (67)
5	**2-NH2PEG**	10.8	0.57	4.1	228 (1.8)	137 (62)
6	**2-NH2PEG**	12.8	1.43	10.2	234 (2.0)	138 (61)
7	**3-NH2PEG**	12.8	8.23	58.8	111 (1.8)	136 (67)
8	**4-NH2PEG**	12.8	3.08	22.0	173 (2.0)	138 (65)
9	**3^{SO3}-pyr**	12.8	8.35	59.7	4.43 (2.5)	127 (76)
10	**4^{SO3}-pyr**	12.8	1.64	11.7	18.7 (1.8)	132 (80)

Polymerization conditions: 50 µmol precatalyst loading, 90 °C, 30 bar, 1 h, 100 mL of oxygen free water, 3.0 g sodium dodecyl sulfate (SDS), 1000 rpm pitched blade stirrer. § TOF given in units of 10^3 mol [C_2H_4] mol^{-1} [Ni] h^{-1}. * Determined by SEC in 1,2-dichlorobenzene at 160 °C (0.5 mL min^{-1}) *via* universal calibration versus narrow polystyrene standards. ** Determined by DSC (10 K min^{-1}), second heating cycle.

Supplementary IR spectra of polymers

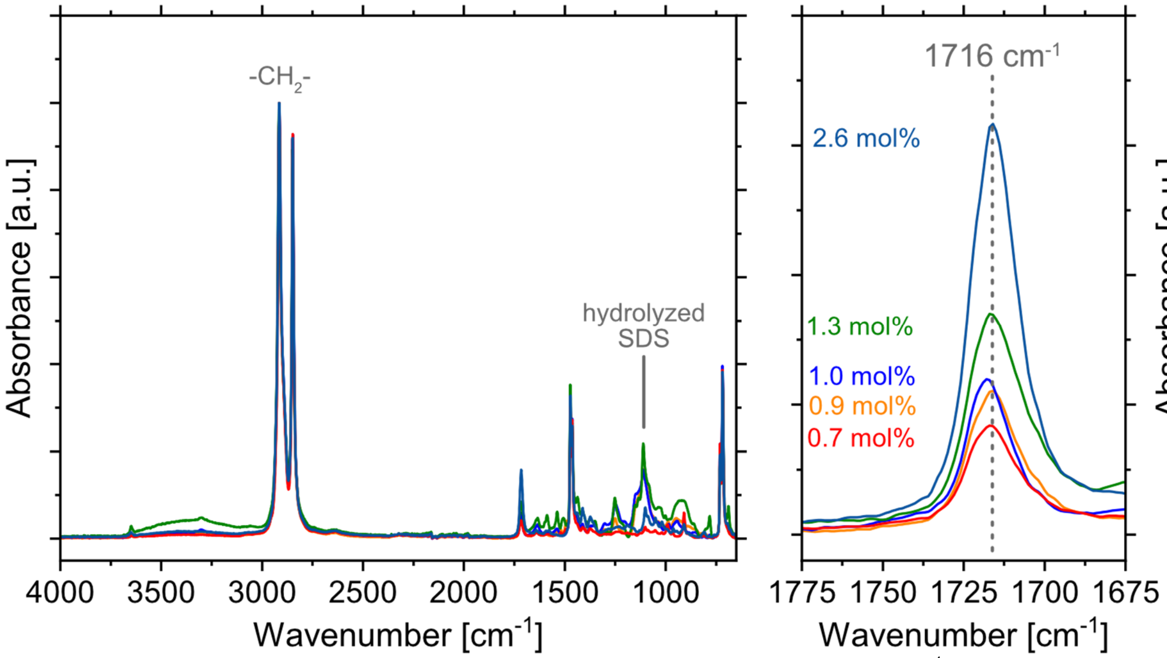

Figure S11. ATR-IR spectra (left) with details of carbonyl region (right, 1675 – 1775 cm^{-1}) of keto-PEs with different carbonyl contents obtained from aqueous nonalternating copolymerization of ethylene and CO (*cf.* Tables S1 and S2).

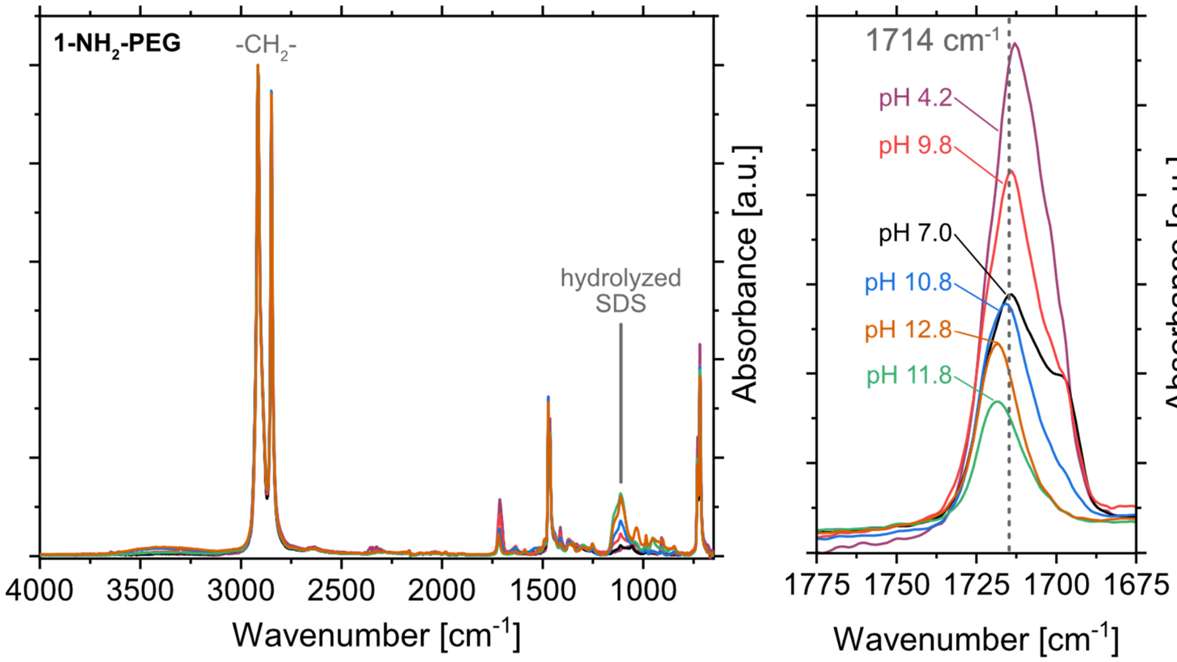

Figure S12. ATR-IR spectra (left) with details of carbonyl region (right, 1675 – 1775 cm^{-1}) of keto-PEs obtained from aqueous nonalternating copolymerization of ethylene and CO with catalyst precursor **1-NH$_2$PEG** at different pH values (Table S2, entries 2 – 7). Note that different ratios of isolated C=O and more alternating C=O motifs are indicated by broadening or a shoulder of the C=O absorption band.

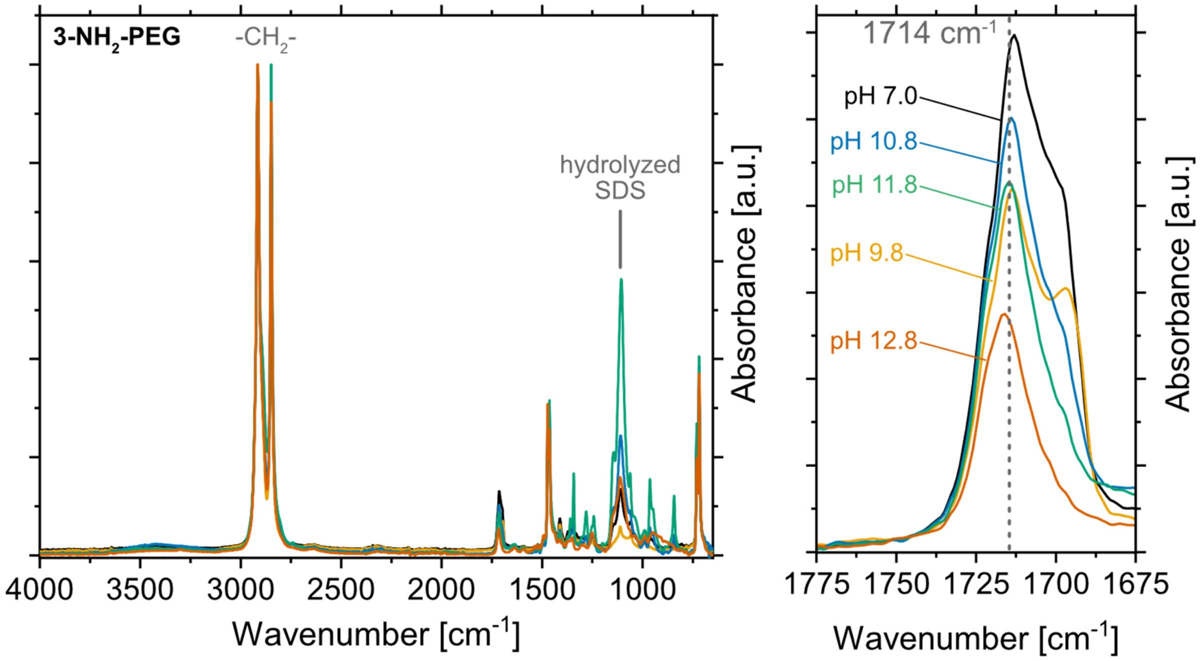

Figure S13. ATR-IR spectra (left) with details of carbonyl region (right, 1675 – 1775 cm^{-1}) of keto-PEs obtained from aqueous nonalternating copolymerization of ethylene and CO with catalyst precursor **3-NH$_2$PEG** at different pH values (Table S2, entries 12 – 16). Note that different ratios of isolated C=O and more alternating C=O motifs are indicated by broadening or a shoulder of the C=O absorption band.

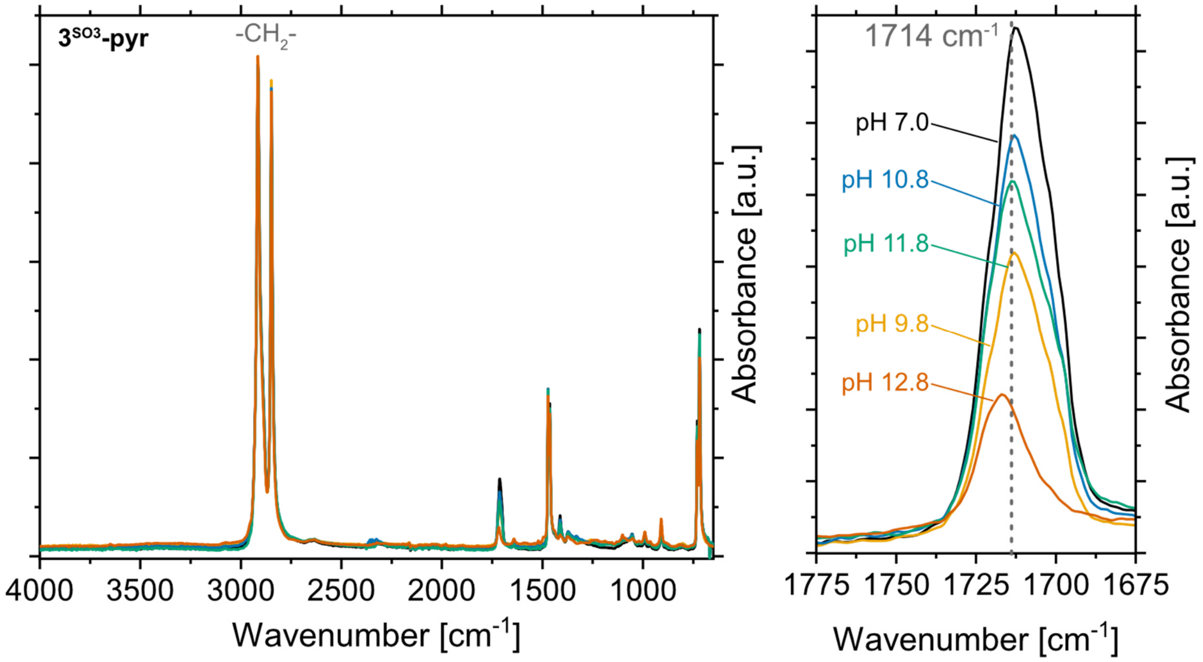

Figure S14. ATR-IR spectra (left) with details of carbonyl region (right, 1675 – 1775 cm^{-1}) of keto-PEs obtained from aqueous nonalternating copolymerization of ethylene and CO with catalyst precursor **3^{SO3}-pyr** at different pH values (Table S2, entries 26 – 30). Note that different ratios of isolated C=O and more alternating C=O motifs are indicated by broadening or a shoulder of the C=O absorption band.

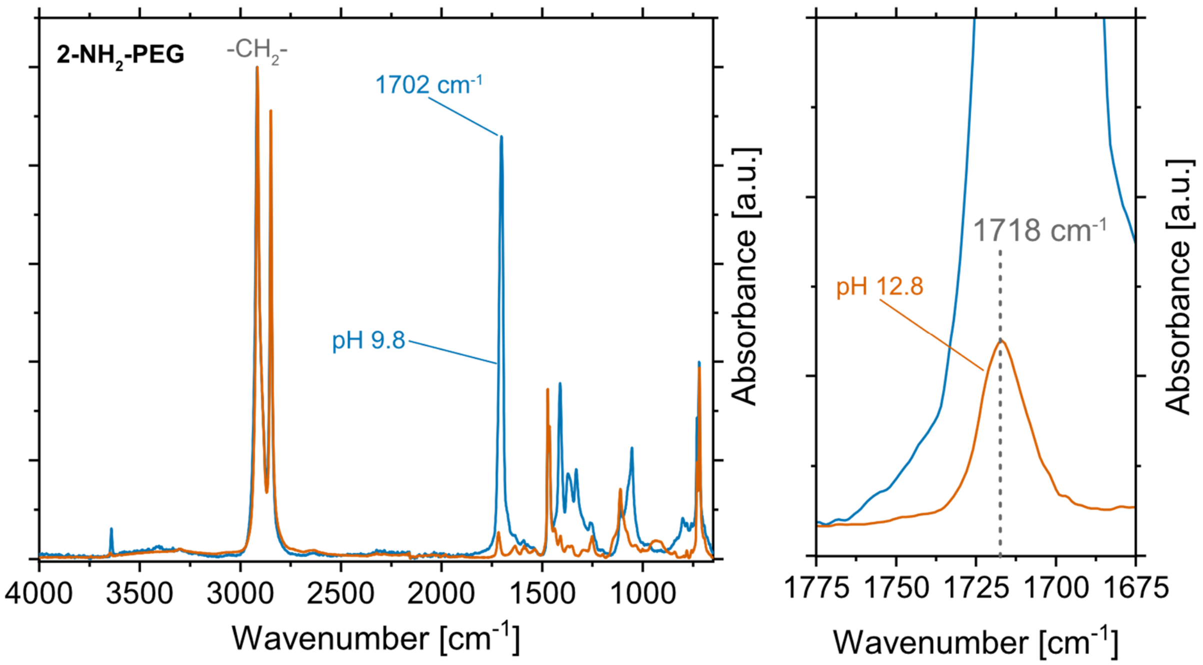

Figure S15. ATR-IR spectra (left) with details of carbonyl region (right, 1675 – 1775 cm^{-1}) of copolymers obtained from aqueous nonalternating copolymerization of ethylene and CO with catalyst precursor **2-NH2PEG** at different pH values (Table S2, entries 9 and 16).

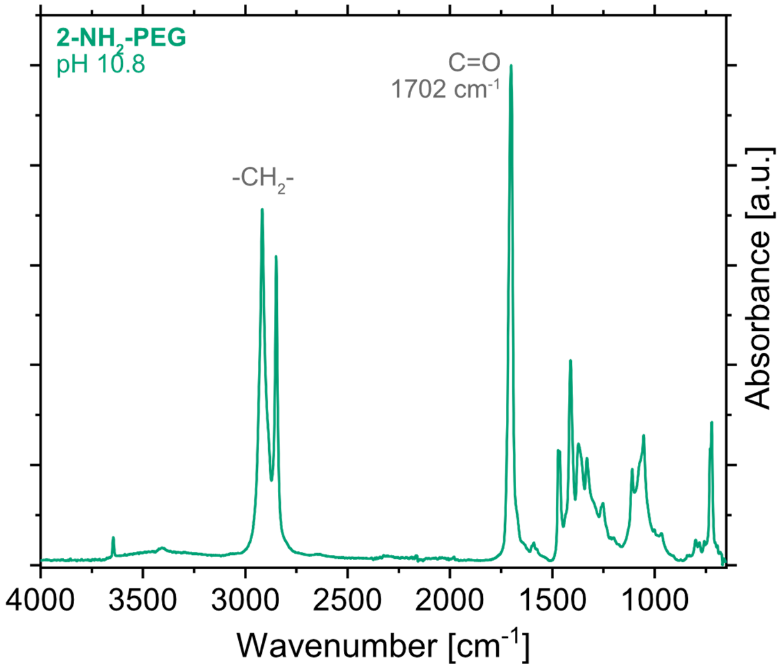

Figure S16. ATR-IR spectrum of a copolymer obtained from aqueous nonalternating copolymerization of ethylene and CO with catalyst precursor **2-NH2PEG** at pH 10.8 (Table S2, entry 14).

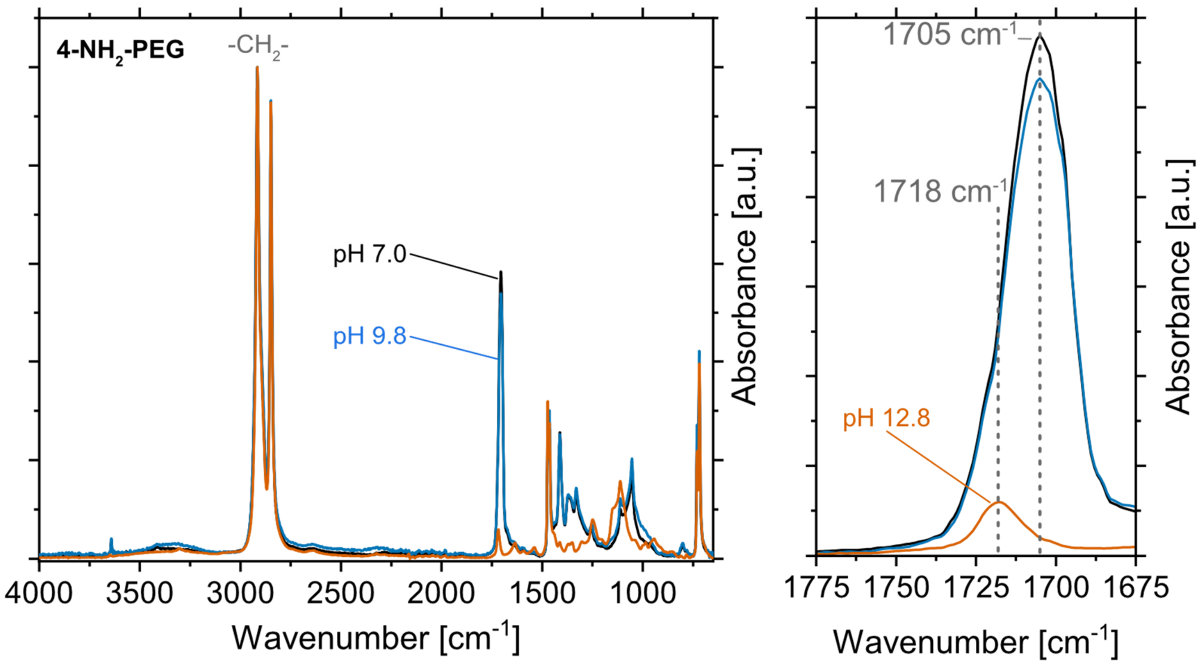

Figure S17. ATR-IR spectra (left) with details of carbonyl region (right, 1675 – 1775 cm^{-1}) of copolymers obtained from aqueous nonalternating copolymerization of ethylene and CO with catalyst precursor **4-NH2PEG** at different pH values (Table S2, entries 17, 18 and 20).

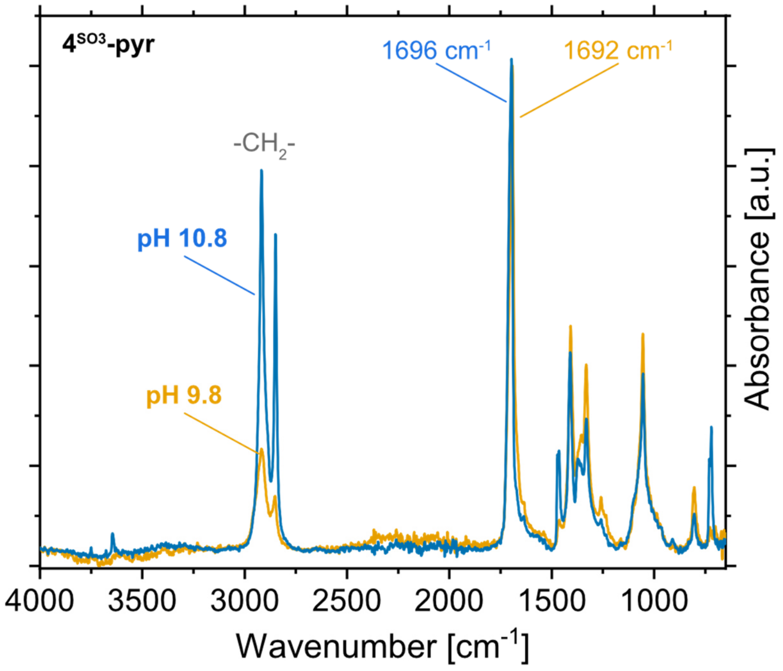

Figure S18. ATR-IR spectra of copolymers obtained from aqueous nonalternating copolymerization of ethylene and CO with catalyst precursor **4^{SO3}-pyr** at 'low' pH values (Table S2, entries 27 and 28).

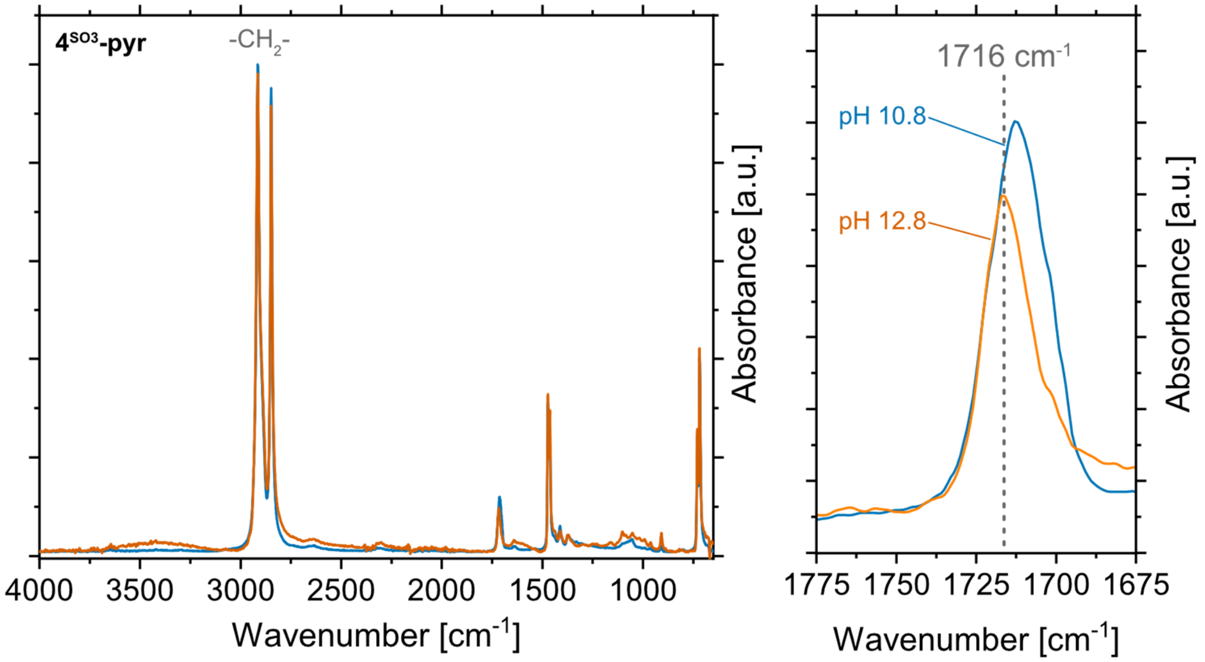

Figure S19. ATR-IR spectra (left) with details of carbonyl region (right, 1675 – 1775 cm^{-1}) of keto-PEs obtained from aqueous nonalternating copolymerization of ethylene and CO with catalyst precursor **4^{SO3}-pyr** at high pH values (Table S2, entries 29 and 30). Note that different ratios of isolated C=O and more alternating C=O motifs are indicated by broadening or a shoulder of the C=O absorption band.

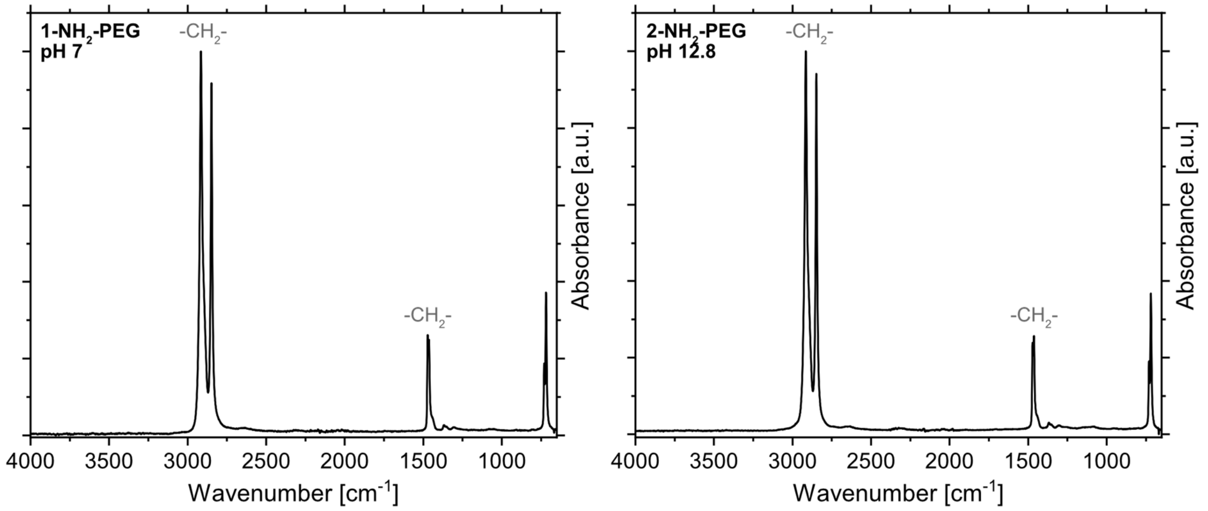

Figure S20. ATR-IR spectra of PE homopolymers obtained by aqueous copolymerization with precatalysts **1-NH$_2$PEG** at pH 7 (left; Table S1, entry 1) and **2-NH$_2$PEG** at pH 12.8 (right; Table S1, entry 6).

Supplementary NMR data of polymers

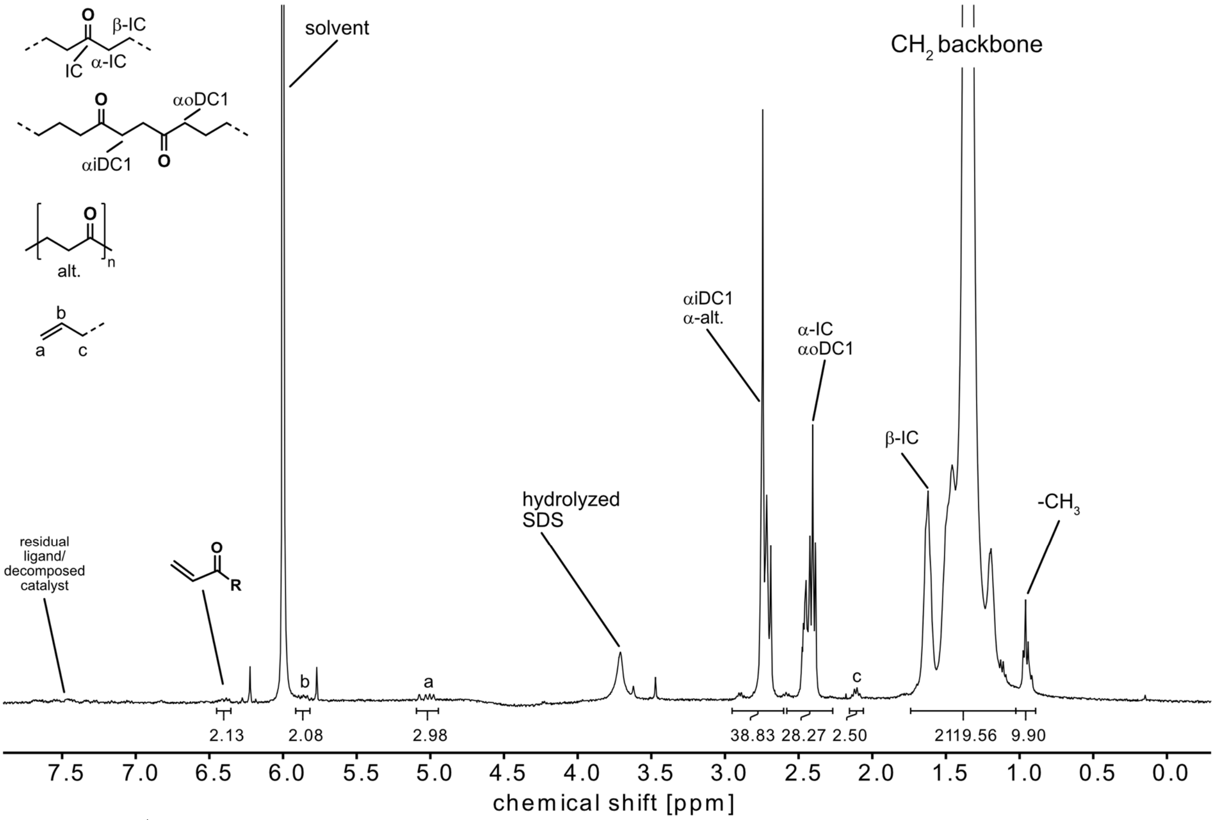

Figure S21. ^{1}H NMR spectrum (400 MHz, 383 K, $C_2D_2Cl_4$) of a keto-PE obtained from aqueous copolymerization at pH 4.2 employing complex **1-NH2PEG** (Table S2, entry 2).

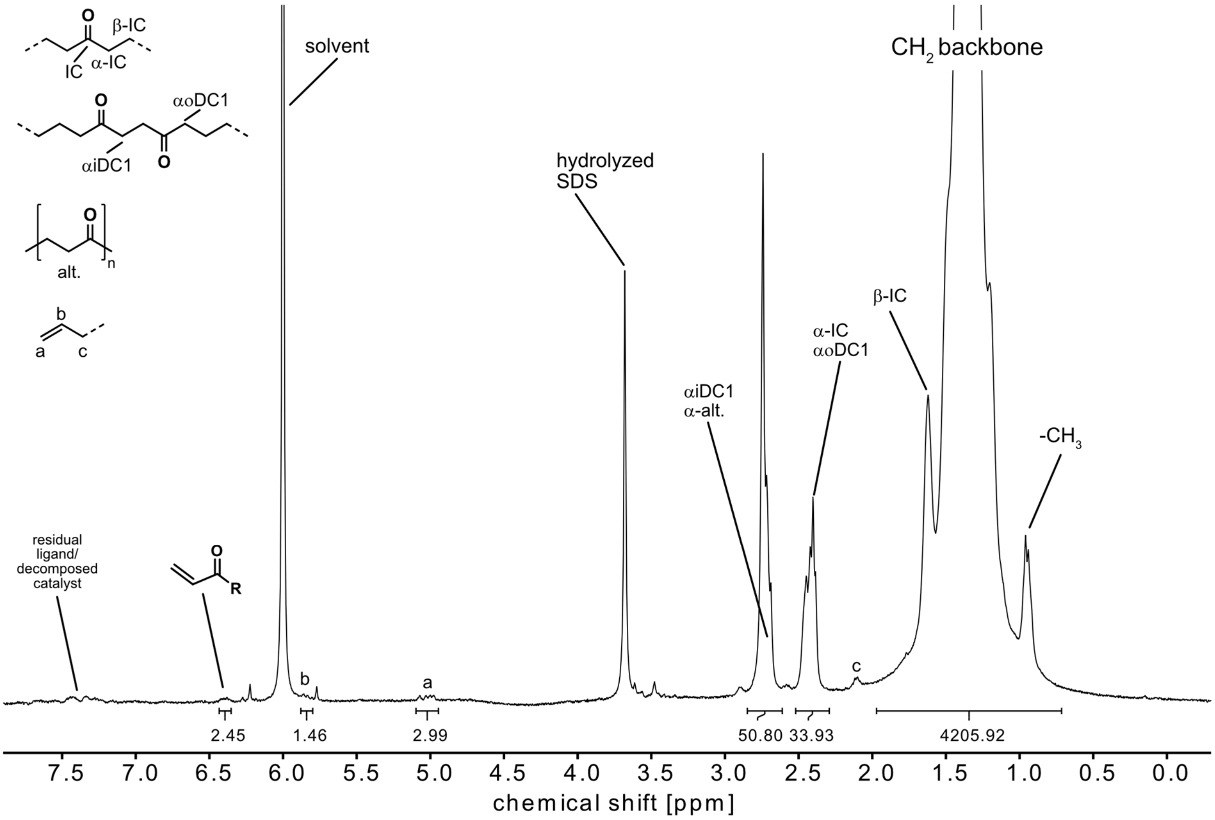

Figure S22. ^{1}H NMR spectrum (400 MHz, 383 K, $C_2D_2Cl_4$) of a keto-PE obtained from aqueous copolymerization at pH 7 employing complex **1-NH2PEG** (Table S2, entry 3).

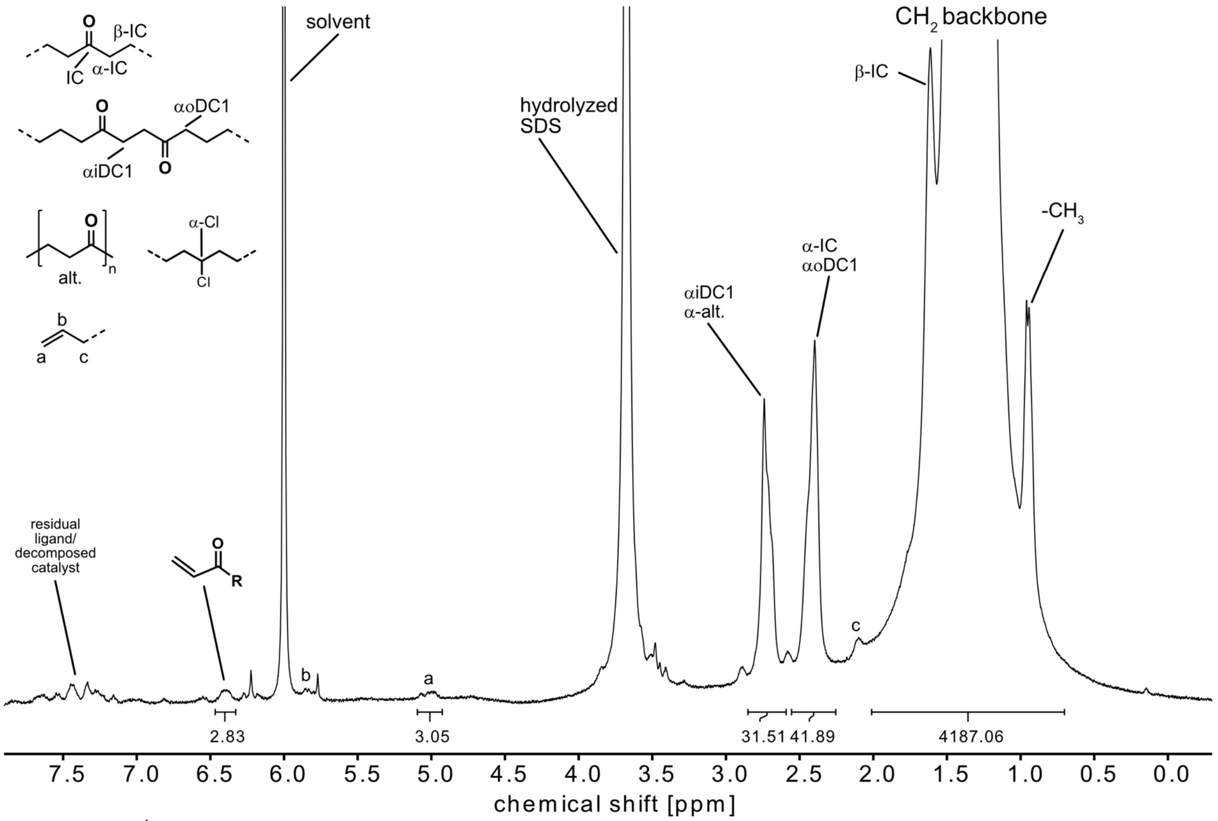

Figure S23. ^{1}H NMR spectrum (400 MHz, 383 K, $C_2D_2Cl_4$) of a keto-PE obtained from aqueous copolymerization at pH 10.8 employing complex **1-NH₂PEG** (Table S2, entry 5).

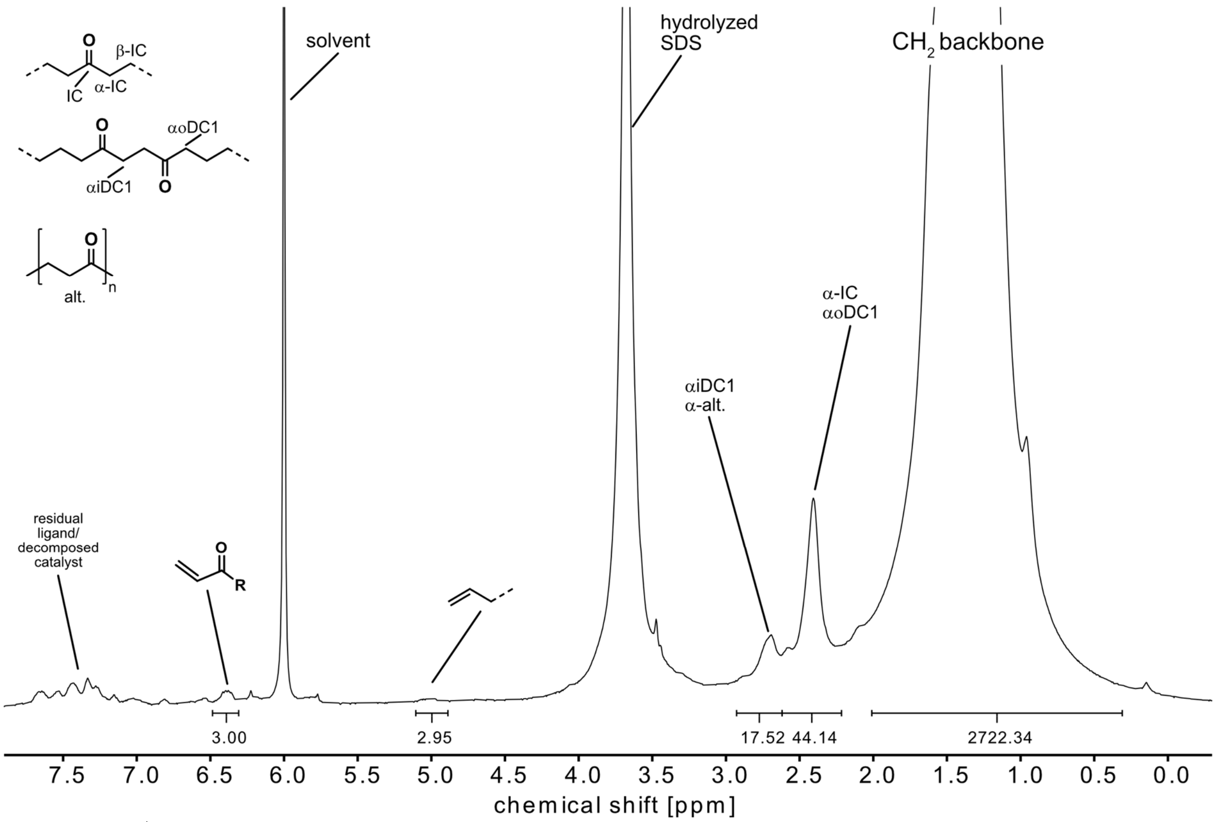

Figure S24. ^{1}H NMR spectrum (400 MHz, 383 K, $C_2D_2Cl_4$) of a keto-PE obtained from aqueous copolymerization at pH 12.8 employing complex **1-NH₂PEG** (Table S2, entry 7).

Table S4. Ratio of α,β-unsaturated carbonyl endgroups formed by deprotonation of carbonyl intermediates vs. unfunctionalized olefinic endgroups from chain transfer by β-H elimination in dependence of the pH/OH^- concentration.

#	Cat.	pH	**I-1** α,β-unsaturated C=O [6.45 ppm, 1H]	**I-2** H_2C=CH- [5.00 ppm, 2H]	**Ratio: I-1/(0.5*I-2)**
1	**1-NH_2PEG**	4.2	2.13	2.98	1.43
2	**1-NH_2PEG**	7	2.45	3.05	1.64
3	**1-NH_2PEG**	10.8	2.83	2.99	1.85
4	**1-NH_2PEG**	12.8	3.00	2.95	2.02

Ratios calculated by ^{1}H NMR spectroscopy (cf. Figures S21 – S24) via integration of the proton resonance of α,β-unsaturated carbonyl endgroups (~ 6.45 ppm) and comparing to the integral value of unfunctionalized olefinic endgroups (~ 5.00 ppm). Integral values are corrected for proton multiplicity.

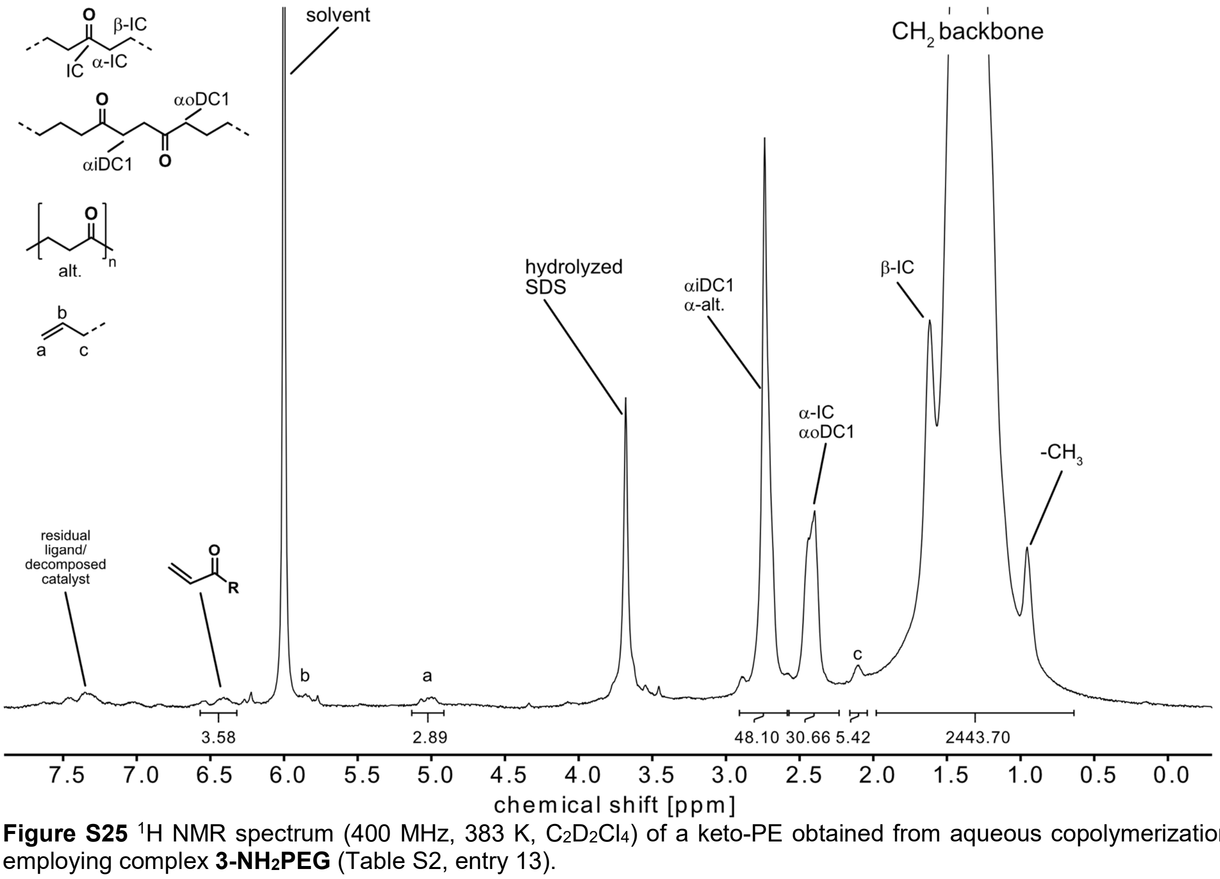

Figure S25 ^{1}H NMR spectrum (400 MHz, 383 K, $C_2D_2Cl_4$) of a keto-PE obtained from aqueous copolymerization at pH 9.8 employing complex **3-NH_2PEG** (Table S2, entry 13).

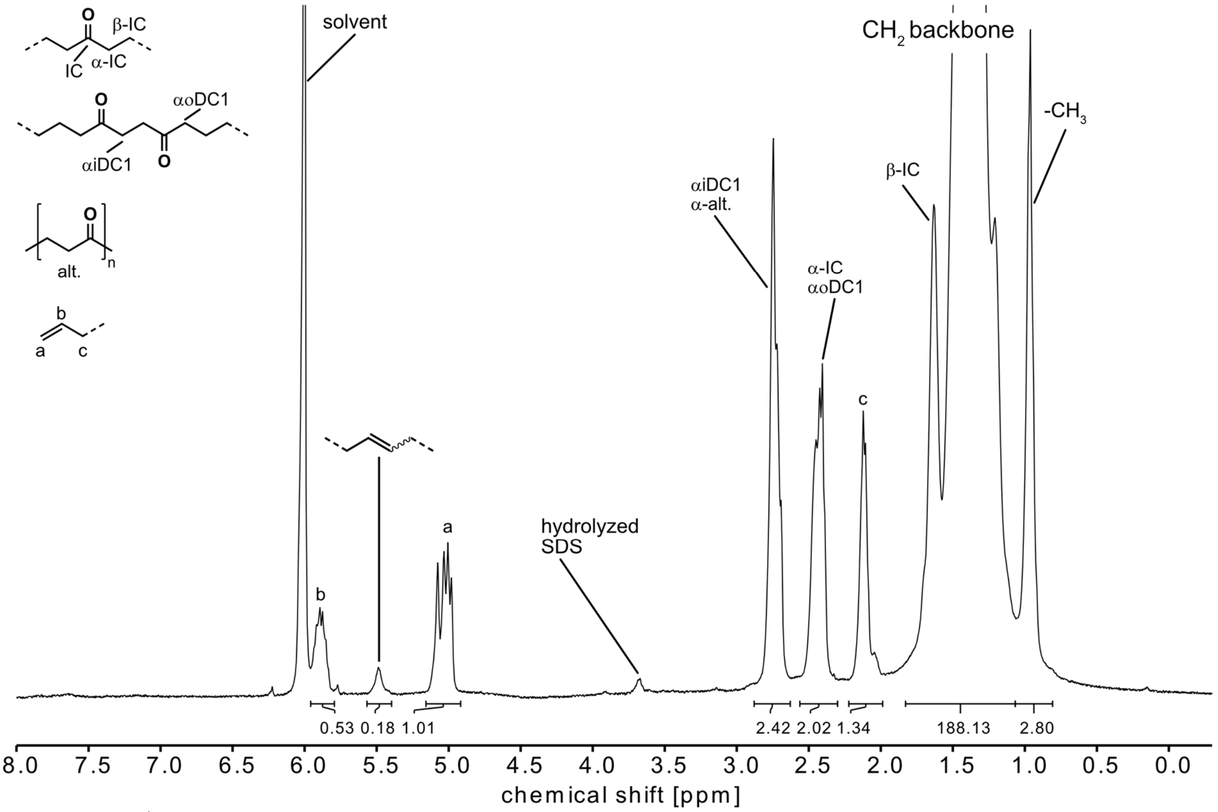

Figure S26. ^1H NMR spectrum (400 MHz, 383 K, $C_2D_2Cl_4$) of a keto-PE obtained from aqueous copolymerization at pH 9.8 employing complex $\mathbf{3^{SO3}}$**-pyr** (Table S2, entry 22).

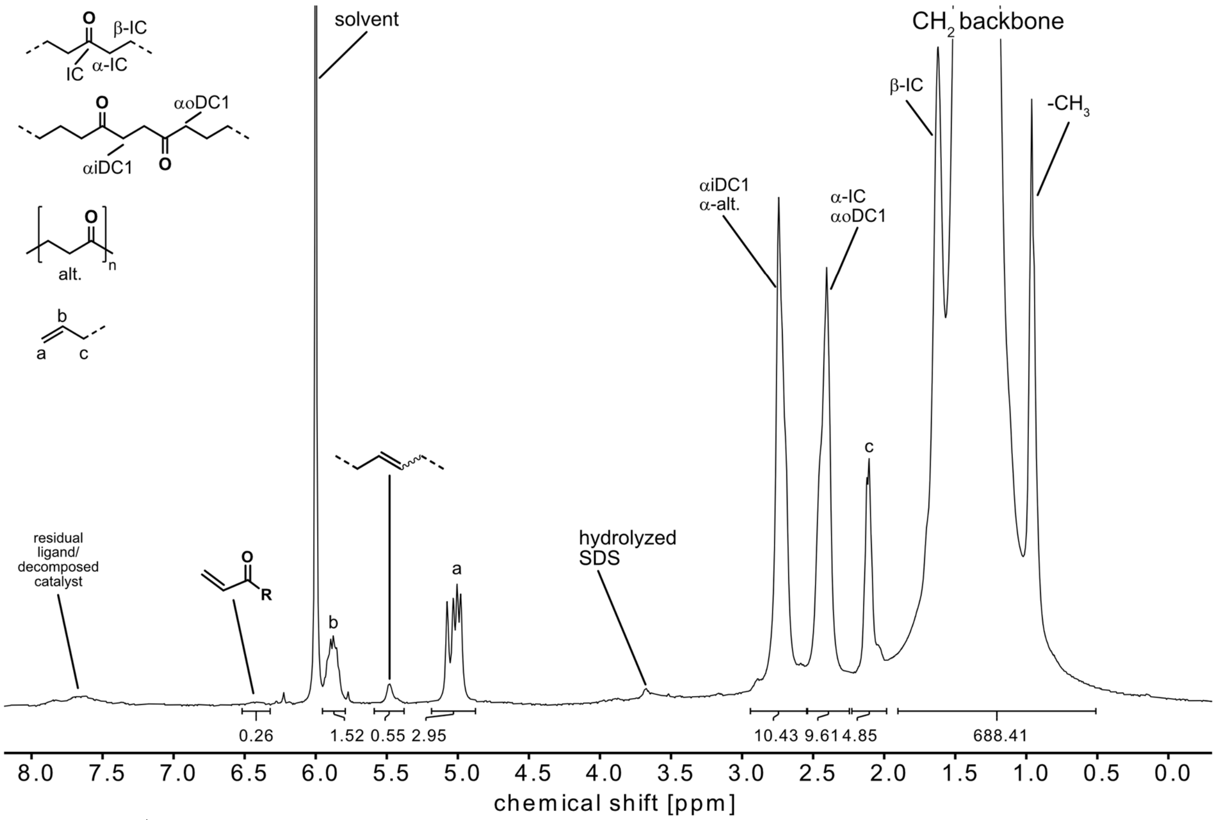

Figure S27. ^1H NMR spectrum (400 MHz, 383 K, $C_2D_2Cl_4$) of a keto-PE obtained from aqueous copolymerization at pH 11.8 employing complex $\mathbf{3^{SO3}}$**-pyr** (Table S2, entry 24).

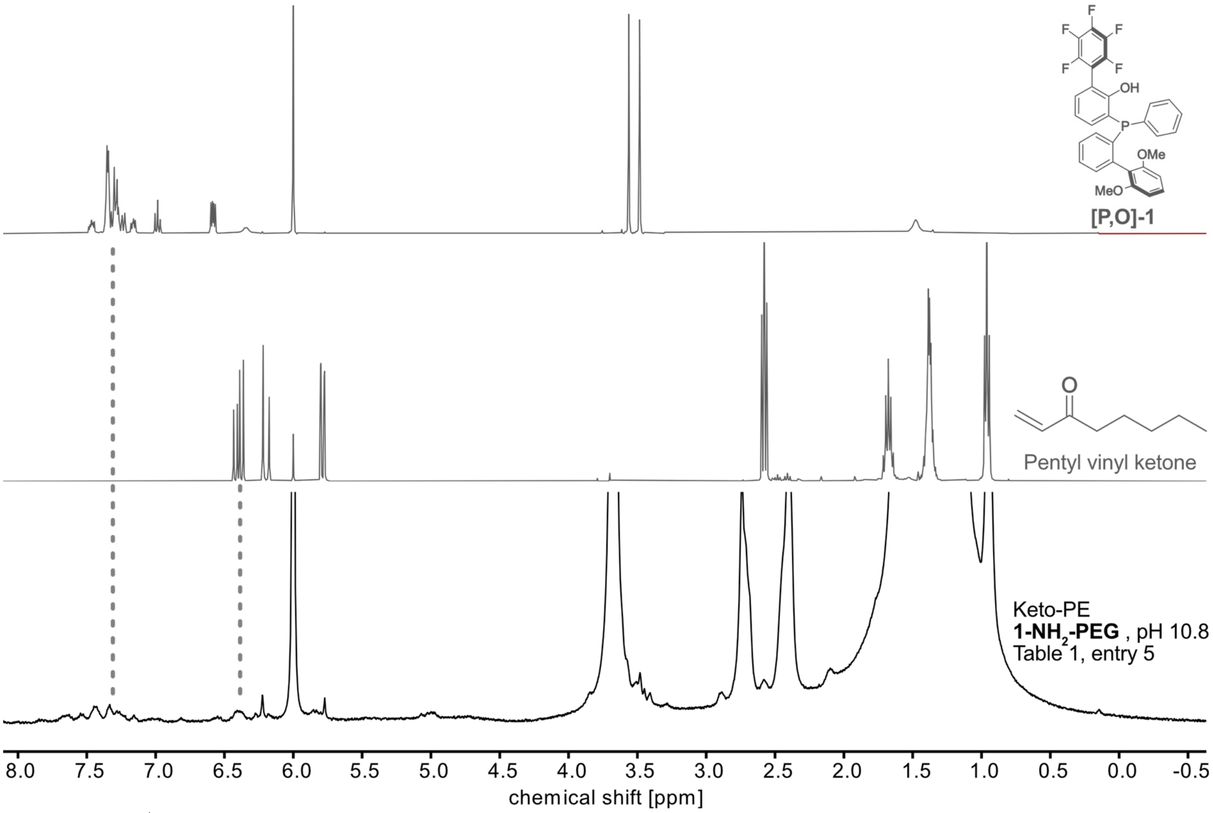

Figure S28. ^{1}H NMR spectra (400 MHz, 383 K, $C_2D_2Cl_4$) of a keto-PE obtained from aqueous copolymerization at pH 10.8 employing complex **1-NH₂PEG** (Table S2, entry 5. Bottom), and of phosphino phenol **[P,O]-1** (top) and pentyl vinyl ketone (center) for comparison.

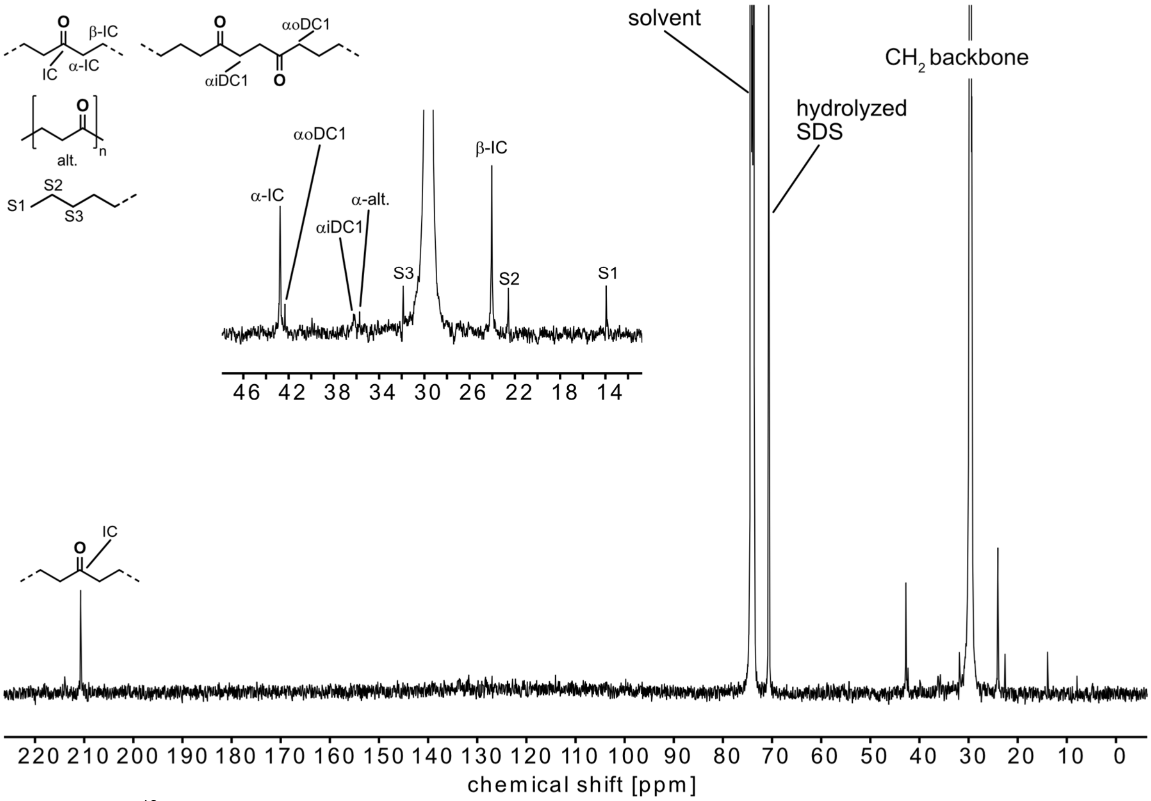

Figure S29. ^{13}C NMR spectrum (101 MHz, 383 K, $C_2D_2Cl_4$) of a keto-PE obtained from aqueous copolymerization at pH 12.8 employing complex **1-NH₂PEG** (Table S2, entry 7).

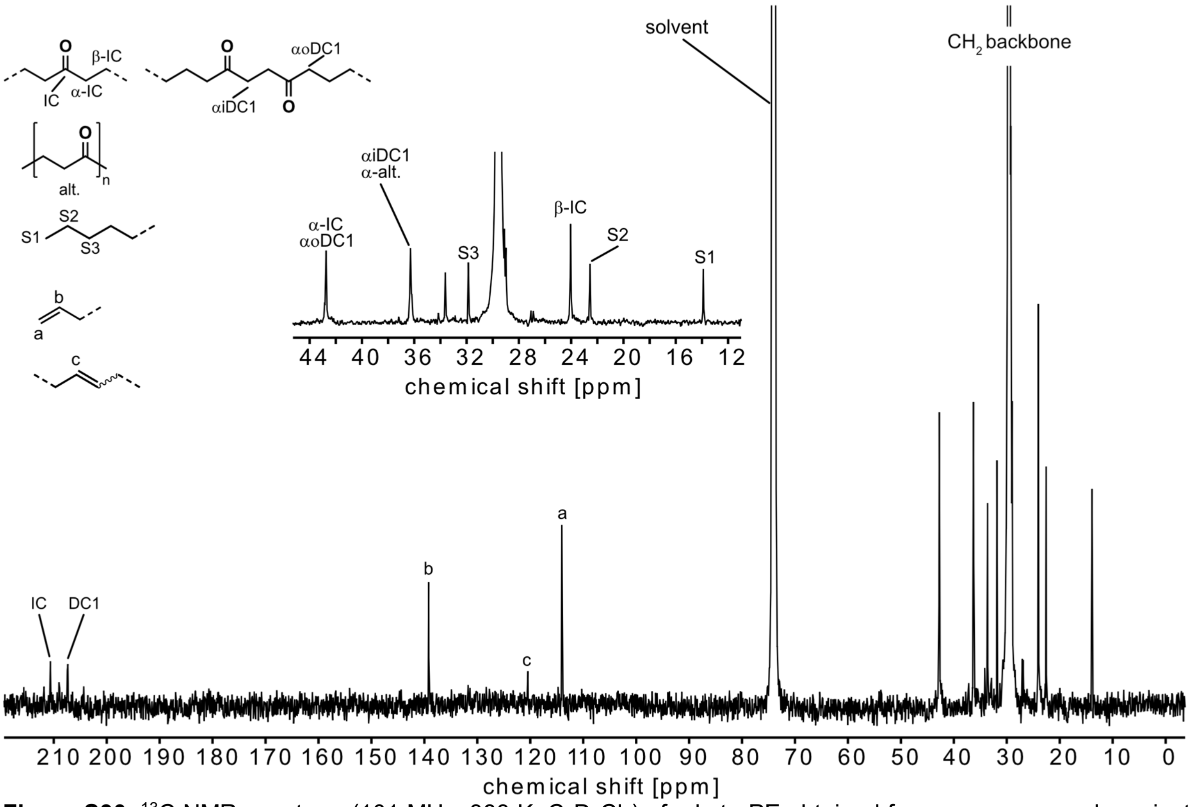

Figure S30. ^{13}C NMR spectrum (101 MHz, 383 K, $C_2D_2Cl_4$) of a keto-PE obtained from aqueous copolymerization at pH 11.8 employing complex **3^{SO3}-pyr** (Table S2, entry 24).

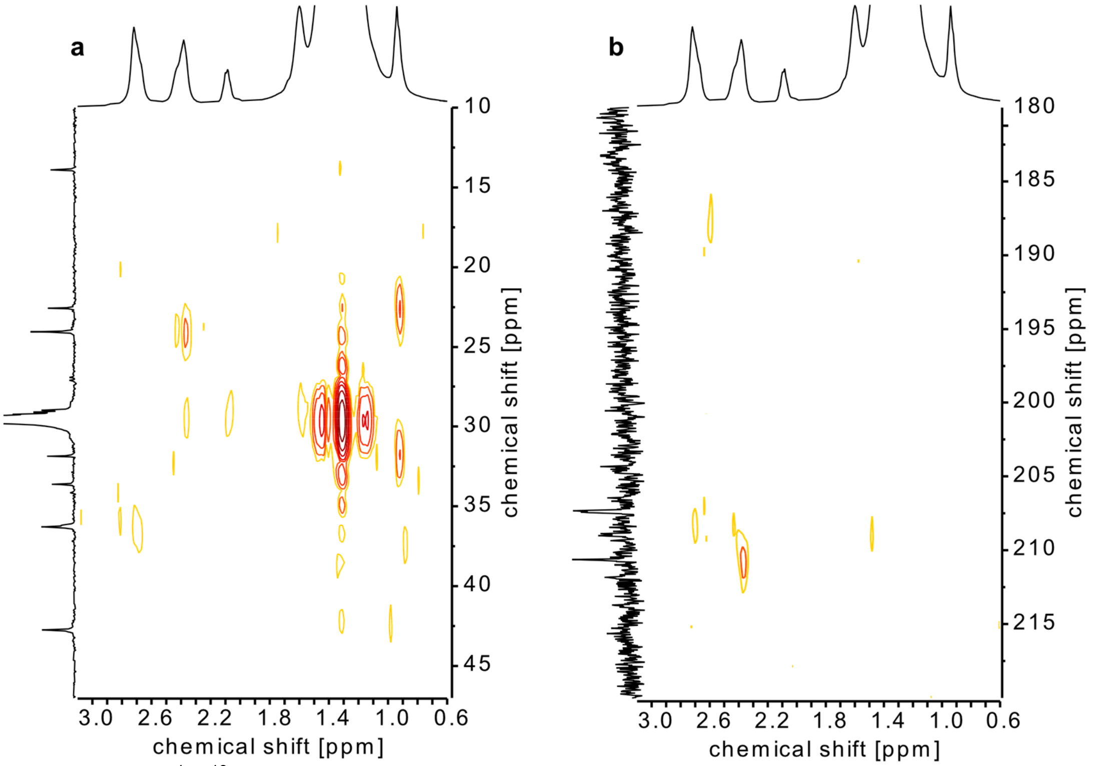

Figure S31. 2D ^{1}H-^{13}C HMBC NMR spectra (383 K, $C_2D_2Cl_4$) of a keto PE obtained from aqueous copolymerization at pH 11.8 employing complex **3^{SO3}-pyr** (Table S2, entry 24). **a**: ^{1}H-^{13}C HMBC, alkyl region (10 – 47 ppm, 0.6 – 3.1 ppm). **b**: ^{1}H-^{13}C HMBC of carbonyls (180 – 220 ppm, 0.6 – 3.1 ppm).

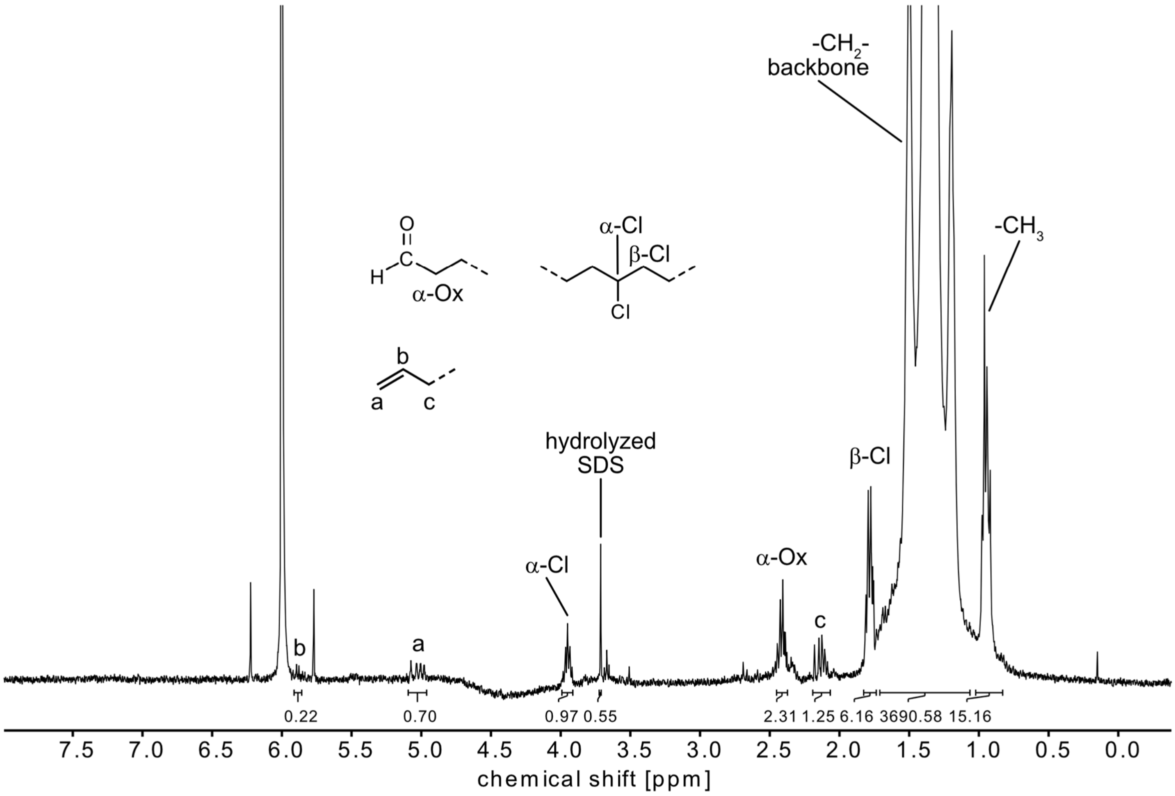

Figure S32. ^{1}H NMR spectrum (400 MHz, 383 K, $C_2D_2Cl_4$) of a PE homopolymer obtained from aqueous polymerization employing complex **1-NH$_2$PEG** (Table S3, entry 3). Note the occurrence of small extents of backbone oxidation and chlorination[6] by the solvent due to the long high-temperature measurement.

SEC traces of polymers

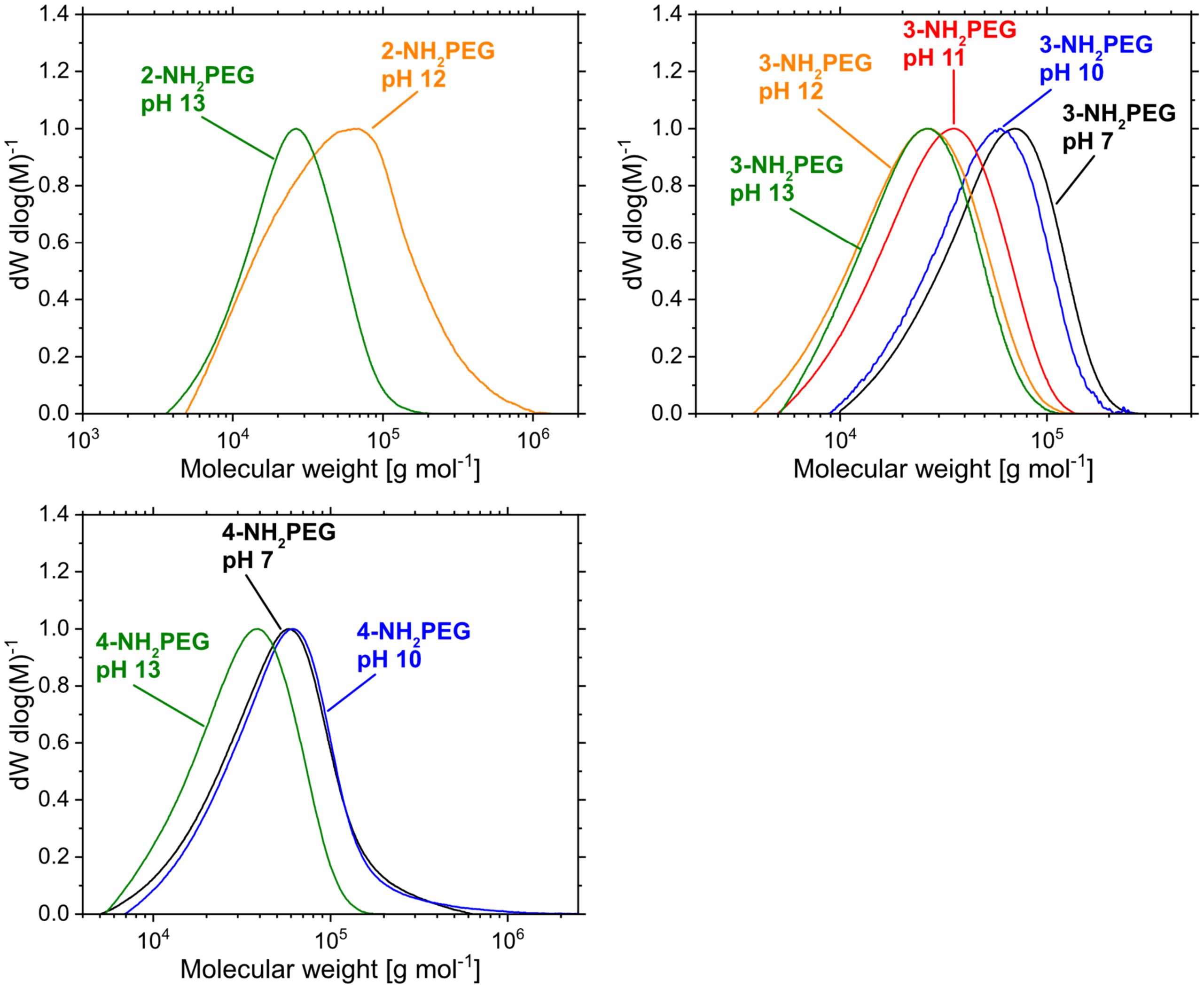

Figure S33. SEC traces (160 °C, 1,2-dichlorobenzene, 0.5 mL min^{-1}, universal calibration against PS standards) of ethylene-CO copolymers obtained from aqueous copolymerizations at different pH, employing complexes bearing NH_2-PEG labile ligands (Table S2, entries 8 – 20).

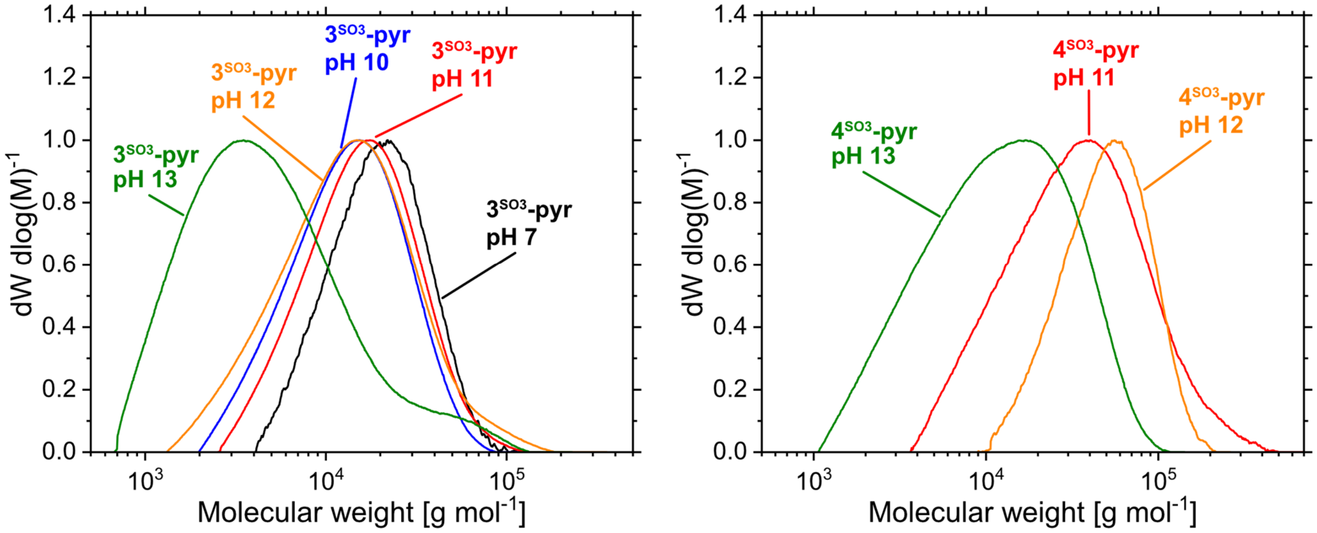

Figure S34. SEC traces (160 °C, 1,2-dichlorobenzene, 0.5 mL min^{-1}, universal calibration against PS standards) of ethylene-CO copolymers obtained from aqueous copolymerizations at different pH, employing hydrophilic catalysts bearing –SO_3Na groups (Table S2, entries 21 – 30).

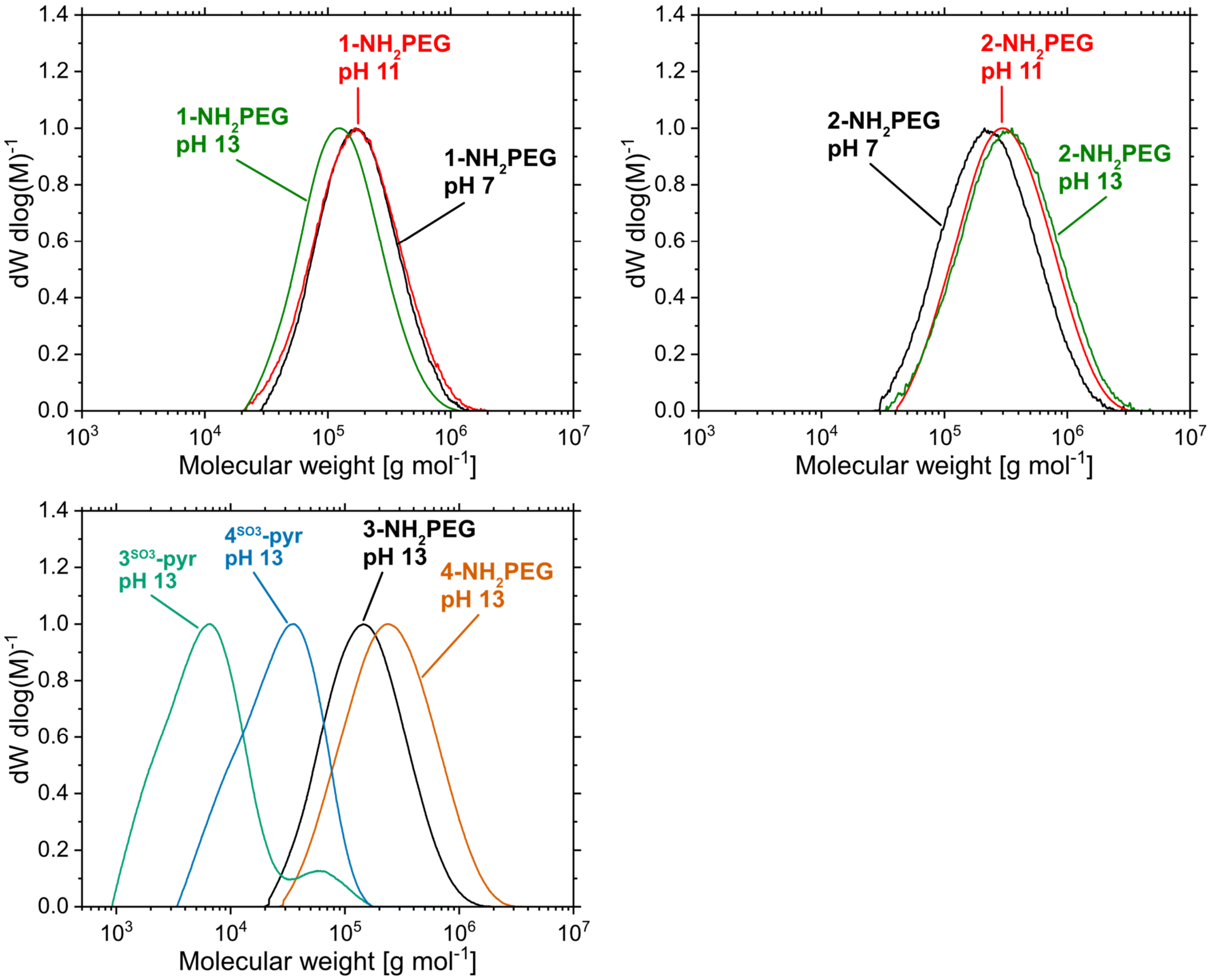

Figure S35. SEC traces (160 °C, 1,2-dichlorobenzene, 0.5 mL min^{-1}, universal calibration against PS standards) of PE homopolymers obtained from aqueous ethylene homopolymerizations at different pH, employing complexes bearing NH_2-PEG labile ligands and hydrophilic catalysts bearing –SO_3Na groups (*cf.* Table S3).

Thermal properties from differential scanning calorimetry (DSC)

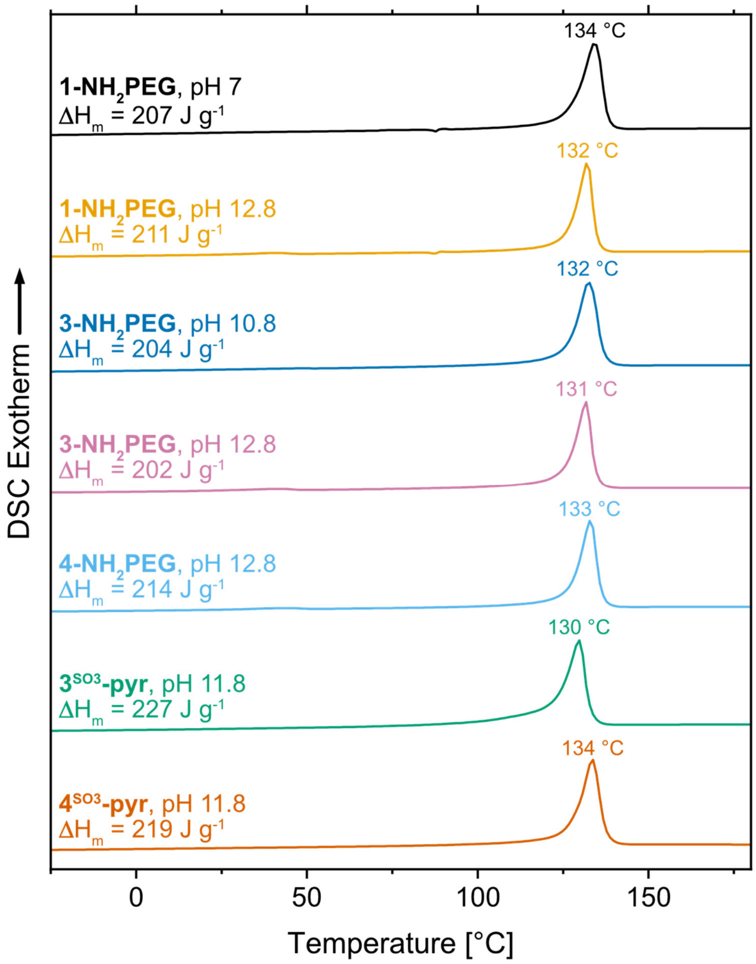

Figure S36. Representative differential scanning calorimetry (DSC) traces (second heating, 10 K min^{-1}) of selected keto-PE polymers obtained from aqueous copolymerization with different catalysts and under different conditions (cf. Table S2).

Dynamic light scattering (DLS) of particle dispersions

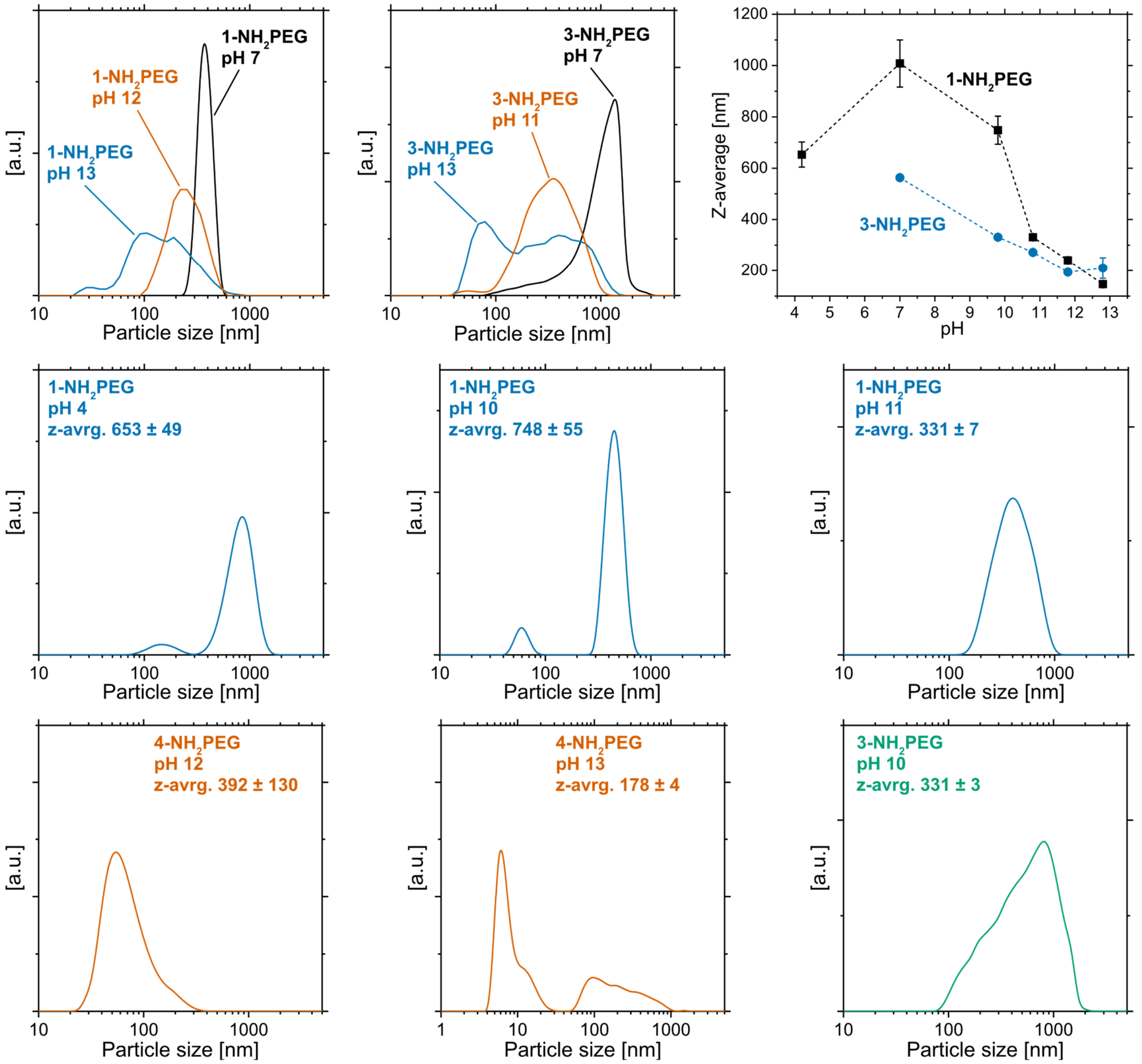

Figure S37. Exemplary dynamic light scattering data of selected keto-PE dispersion obtained from catalytic aqueous copolymerization at different pH, employing precatalysts **1-NH$_2$PEG, 3-NH$_2$PEG** and **4-NH$_2$PEG**.

Transmission electron microscopy (TEM)

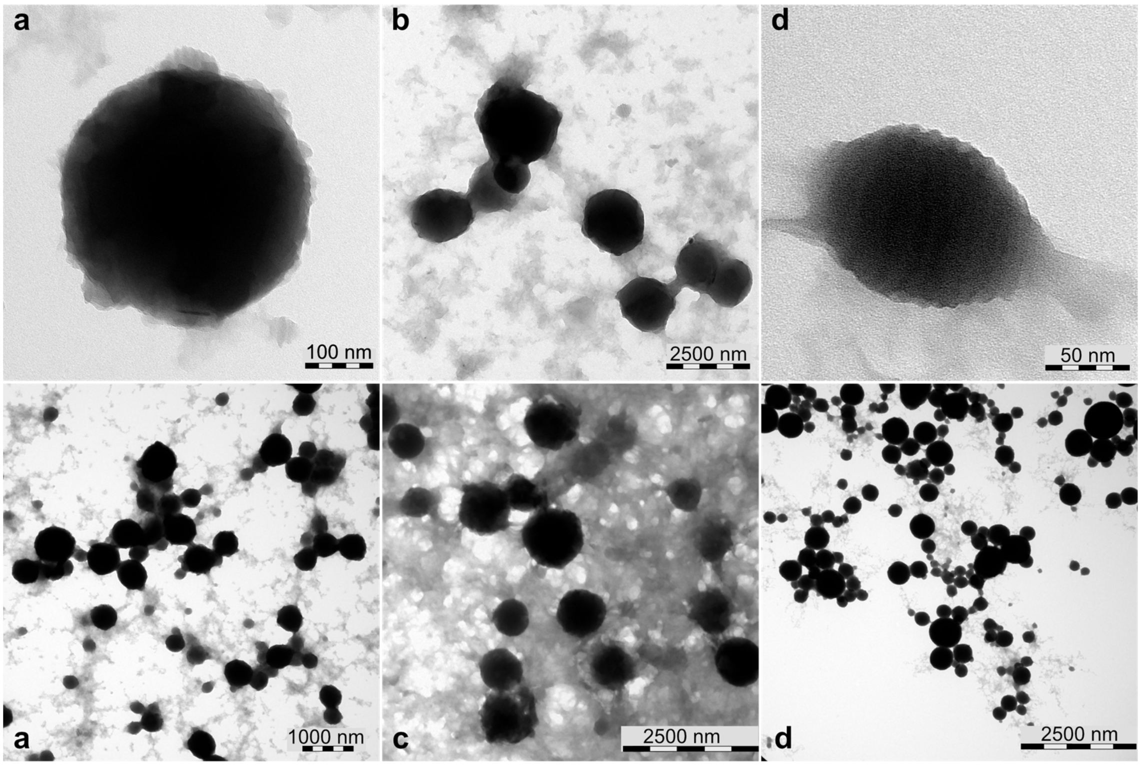

Figure S38. Transmission electron microscopy (TEM) of keto-PE particles obtained from aqueous catalytic copolymerization employing: **a: 1-NH$_2$PEG**, pH 7 (Table S2 entry 3). **b: 1-NH$_2$PEG**, pH 11 (Table S2, entry 5). **c: 1-NH$_2$PEG**, pH 12 (Table S2, entry 6). **d: 3-NH$_2$PEG**, pH 7 (Table S2, entry 12).

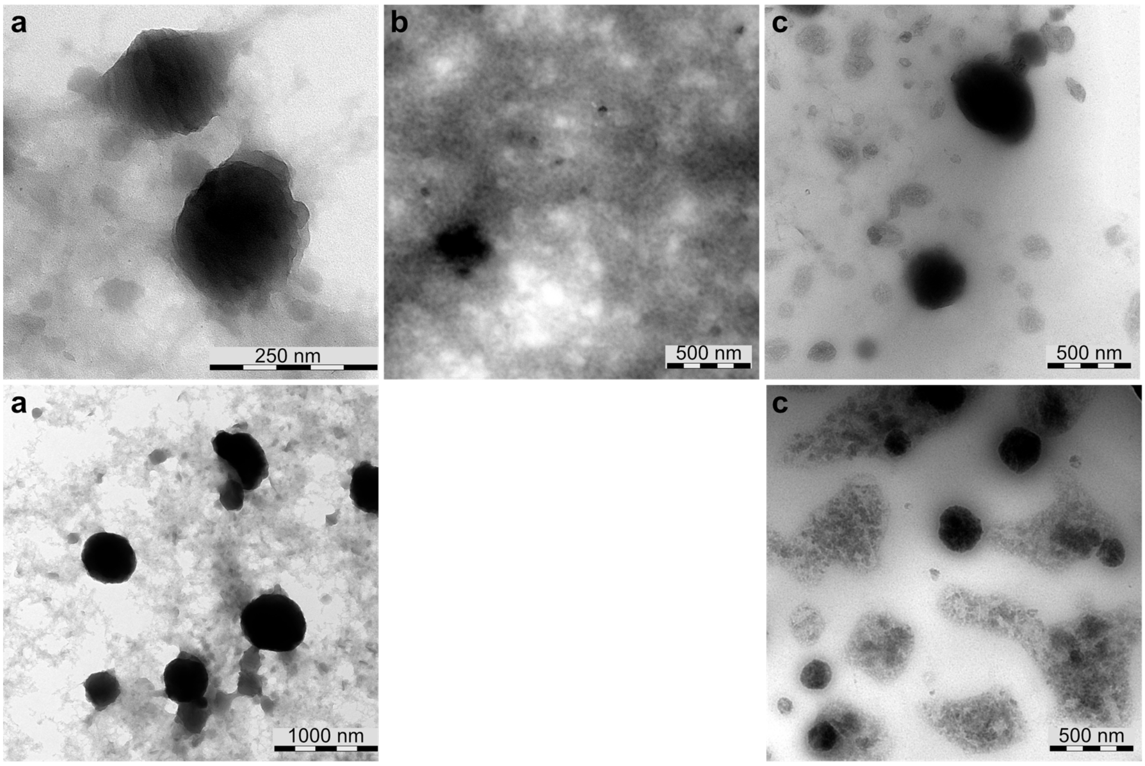

Figure S39. Transmission electron microscopy (TEM) of keto-PE particles obtained from aqueous catalytic copolymerization employing: **a: 3-NH$_2$PEG**, pH 12 (Table S2, entry 15). **b: 4-NH$_2$PEG**, pH 7 (Table S2, entry 17). **c: 4-NH$_2$PEG**, pH 11 (Table S2, entry 19).

Keto-PE film formation from aqueous particle dispersions

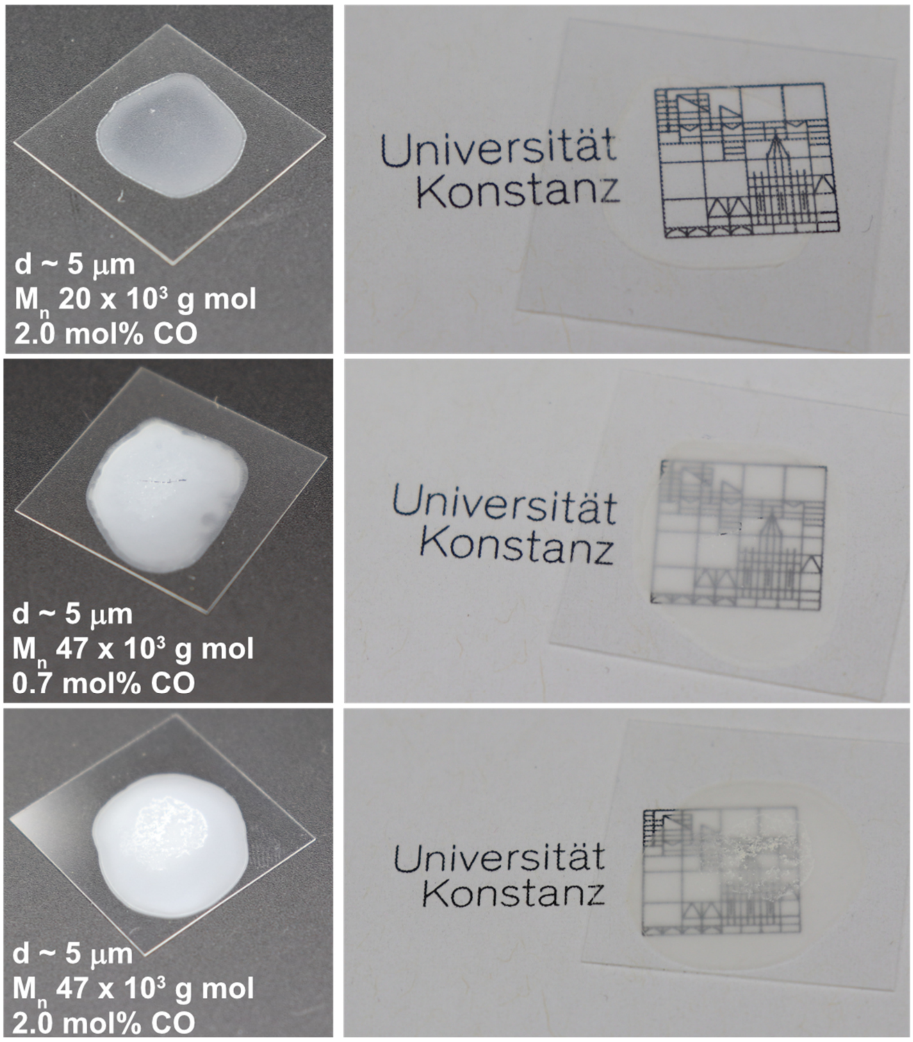

Figure S40. Keto-PE films obtained by dropcasting of dialyzed keto-PE particle dispersions from aqueous catalytic copolymerization.

III. Supporting References

[1] Göttker-Schnetmann, I.; Mecking, S. A Practical Synthesis of [(tmeda)Ni(CH_3)$_2$], Isotopically Labeled [(tmeda)Ni($^{13}CH_3$)$_2$], and Neutral Chelated-Nickel Methyl Complexes. *Organometallics* **2020**, *39* (18), 3433–3440. DOI: 10.1021/acs.organomet.0c00500.

[2] DiCiccio, A. M.; Longo, J. M.; Rodríguez-Calero, G. G.; Coates, G. W. Development of Highly Active and Regioselective Catalysts for the Copolymerization of Epoxides with Cyclic Anhydrides: An Unanticipated Effect of Electronic Variation. *J. Am. Chem. Soc.* **2016**, *138* (22), 7107–7113. DOI: 10.1021/jacs.6b03113.

[3] Poon, P.; Banerjee, A. K.; Bedoya, L.; Sanchez, J.; Laya, M. S. Acid-Washed Bentonite: A New Reagent for the Deprotection of Tetrahydropyranyl Ethers. *J. Chem. Res.* **2011**, *35* (8), 477–479. DOI: 10.3184/174751911X13133127427965.

[4] Morgen, T. O.; Baur, M.; Göttker-Schnetmann, I.; Mecking, S. Photodegradable branched polyethylenes from carbon monoxide copolymerization under benign conditions. *Nat. Commun.* **2020**, *11* (1), 3693. DOI: 10.1038/s41467-020-17542-5.

[5] Ortmann, P.; Wimmer, F. P.; Mecking, S. Long-Spaced Polyketones from ADMET Copolymerizations as Ideal Models for Ethylene/CO Copolymers. *ACS Macro Lett.* **2015**, *4* (7), 704–707. DOI: 10.1021/acsmacrolett.5b00324.

[6] Brandolini, A. J.; Hills, D. D. *NMR spectra of polymers and polymer additives*; CRC Press Taylor & Francis Group, 2000.

[7] Baur, M.; Lin, F.; Morgen, T. O.; Odenwald, L.; Mecking, S. Polyethylene materials with in-chain ketones from nonalternating catalytic copolymerization. *Science* **2021**, *374* (6567), 604–607. DOI: 10.1126/science.abi8183.

[8] Xin, B. S.; Sato, N.; Tanna, A.; Oishi, Y.; Konishi, Y.; Shimizu, F. Nickel Catalyzed Copolymerization of Ethylene and Alkyl Acrylates. *J. Am. Chem. Soc.* **2017**, *139* (10), 3611–3614. DOI: 10.1021/jacs.6b13051.

[9] Zhang, Y.; Mu, H.; Wang, X.; Pan, L.; Li, Y. Elaborate Tuning in Ligand Makes a Big Difference in Catalytic Performance: Bulky Nickel Catalysts for (Co)polymerization of Ethylene with Promising Vinyl Polar Monomers. *ChemCatChem* **2019**, *11* (9), 2329–2340. DOI: 10.1002/cctc.201900265.

[10] Lin, F.; Mecking, S. Hydrophilic Catalysts with High Activity and Stability in the Aqueous Polymerization of Ethylene to High-Molecular-Weight-Polyethylene. *Angew. Chem. Int. Ed.* **2022**, *61* (27), e202203923. DOI: 10.1002/anie.202203923.

[11] Schnitte, M.; Staiger, A.; Casper, L. A.; Mecking, S. Uniform shape monodisperse single chain nanocrystals by living aqueous catalytic polymerization. *Nat. Commun.* **2019**, *10* (1), 2592. DOI: 10.1038/s41467-019-10692-1.
